# *CircTP53*/USP10/p53 signaling Axis as a Novel Regulator of Progression and Prognosis of Head and Neck Squamous Cell Carcinoma

**DOI:** 10.1002/advs.202414961

**Published:** 2025-06-04

**Authors:** Yin Wang, Fen Chang, Zinan Li, Chengcheng Duan, Xiangkai Sun, Siyu Wang, Dongmin Wei, Wenming Li, Ye Qian, Shengda Cao, Juan Zhao, Dapeng Lei

**Affiliations:** ^1^ Department of Otorhinolaryngology Qilu Hospital of Shandong University Jinan Shandong China; ^2^ NHC Key Laboratory of Otorhinolaryngology (Shandong University) Jinan Shandong China

**Keywords:** circTP53, Deubiquitination, HNSCC, p53, USP10

## Abstract

Due to the absence of effective biomarkers, the precision therapy of head and neck squamous cell carcinoma (HNSCC) still faces challenge. TP53 is one of the most frequently mutated genes in human cancers including HNSCC. Although studies on the regulation of TP53 gene and p53 protein have been extensively explored, the association of TP53‐derived circRNAs with HNSCC progression, along with their regulatory mechanisms, remains unknown. This study identifies a novel circRNA derived from TP53 (circTP53), which is upregulated in HNSCC and associated with poor prognosis. It is demonstrated that circTP53 promotes HNSCC progression in vitro and in vivo. Mechanistically, circTP53 interacts with the deubiquitinase USP10, leading to their mutual stabilization, which enhances USP10's deubiquitinating activity on p53, thereby stabilizing p53. Interaction analysis reveals that intron 9 of circTP53 interacts with 100–399AA of USP10. In tumor cells with wild‐type p53, circTP53 suppresses cell viability and inhibits the growth of xenograft tumors, while in tumor cells harboring mutant p53, circTP53 demonstrates the opposite effect, enhancing cell viability and promoting xenograft tumor progression. The identification of circTP53 suggests a new direction for p53 research, and the elucidation of circTP53/USP10/p53 axis may provide a new therapeutic scheme for future precision treatment of HNSCC.

## Graphical abstract

The study identifies a novel circular RNA derived from the TP53 gene (circTP53), which is upregulated in HNSCC and correlates with poor patient prognosis. It demonstrates that circTP53 promotes HNSCC progression by interacting with USP10, stabilizing both proteins, enhancing deubiquitination of p53, and thereby influencing tumor growth, with its effects varying depending on the p53 mutation status in tumor cells.

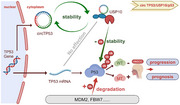

## 1 Introduction

Head and neck squamous cell carcinoma (HNSCC) is the seventh most common type of cancer worldwide. HNSCC represents a heterogeneous group of malignancies arising from the upper aerodigestive tract, which includes subtypes such as the oral cavity, pharynx, and larynx.^[^ ^1^, ^2^ ^]^ Current treatment for HNSCC encompasses traditional approaches such as surgery and chemoradiotherapy, alongside more contemporary strategies including targeted therapy and immunotherapy.^[^ ^3^, ^4^, ^5^ ^]^ Nonetheless, ongoing challenges persist due to the lack of effective biomarkers and therapeutic targets, hampering the advancement of personalized precision treatments.

Circular RNA (*circRNA*) is a class of single‐stranded closed‐loop RNA molecules. Unlike linear RNAs, *circRNAs* lack a 5′ cap or 3′ poly A tail and instead form covalently closed loops through the back‐splicing of pre‐mRNA transcripts.^[^ ^6^ ^]^ This circular structure renders them resistant to exonuclease‐mediated degradation, making them promising candidates for biomarkers and therapeutic targets in various diseases, including cancer.^[^ ^7^, ^8^ ^]^ They exert distinctive functions through diverse mechanisms, including sponging of microRNAs (miRNAs), regulation of gene expression, modulation of signaling pathways, interaction with functional proteins,^[^ ^9^, ^10^ ^]^ and translation of peptides or proteins. The interaction between *circRNAs* and functional proteins has diverse functional implications in physiological and pathological processes.^[^ ^11^, ^12^ ^]^ For example, *circNEIL3*, identified as a TGFβ‐repressive and metastasis‐related *circRNA*, inhibits tumor metastasis by interacting with Y‐box‐binding protein 1 (YBX1) and promoting its degradation.^[^ ^13^ ^]^ *CircPABPC1*, preferentially lost in tumor cells, inhibits metastases in HCC by suppressing cell adhesion and migration via down‐regulation of ITGB1, leading to proteasomal degradation, revealing a novel mechanism of *circRNA* action in cancer therapy.^[^ ^14^ ^]^ The hsa_circ_0 005185 facilitates Otubain 1 (OTUB1)‐mediated deubiquitination of RAB8A, promoting primary cilia regeneration, inhibiting Hedgehog signaling, and suppressing AR activity to slow castration‐resistant prostate cancer progression.^[^ ^15^ ^]^

The *TP53* gene, located on the short arm of chromosome 17, encoding the p53 protein, is the most commonly mutated gene in human cancers including HNSCC.^[^ ^16^ ^]^ As p53 orchestrates diverse cellular processes such as cell cycle arrest, DNA repair, and apoptosis, p53 is frequently regarded as the “guardian of the genome”. Under certain stress condition, p53‐associated signaling pathways and downstream proteins are activated, such as ASPP1 (apoptosis‐stimulating protein of p53), BAX (a regulator of apoptosis), and p21 (proteins regulating the cell cycle).^[^ ^17^ ^]^ Indeed, wtp53 is a crucial tumor suppressor that plays a pivotal role in maintaining genomic integrity.^[^ ^18^, ^19^ ^]^ However, various mutant forms of p53 can promote tumor proliferation through gain‐of‐function (GOF) mechanisms, or they may exert dominant‐negative functions to antagonize the remaining wtp53.^[^ ^20^, ^21^ ^]^ The regulation of p53 by protein stabilization system is linked to tumor progression, involving the modulation of various ubiquitinating and deubiquitinating enzymes, such as MDM2 and USP10.^[^ ^22^, ^23^, ^24^ ^]^ Nonetheless, the current knowledge of p53 protein regulation cannot fully illustrate the diversity of their involvement in biological processes and tumor development. Notably, little is known about the function of *circRNAs* arising from *TP53*.

In this current study, we identified one *circRNA* derived from *TP53* (*circTP53*), which was upregulated in HNSCC tissues and closely correlated with unfavorable clinical prognosis of HNSCC patients. We further demonstrated that *circTP53* promotes the viability of HNSCC cells in *vivo* and in *vitro*. We found that *circTP53* and deubiquitinase USP10 mutually stabilize each other through direct binding. *CircTP53* enhanced the deubiquitinating activity of USP10, which mediated wtp53 and mtp53 stabilization, leading to the progression of cancer. Our findings indicate that *circTP53* plays a crucial role in promoting tumors by binding to USP10, and we further explored the specific binding domains of *circTP53* and USP10. The identification and functional exploration of *circRNAs* derived from *TP53* enhances our comprehension of the diverse and intricate roles of *TP53* in physiological and pathological processes. This may provide new therapeutic options for individualized precision therapy of HNSCC.

## 2 Results

### 2.1 hsa‐circ‐0041947 Is a Novel *circRNA* Derived from *TP53* in HNSCC

**Figure 1:**
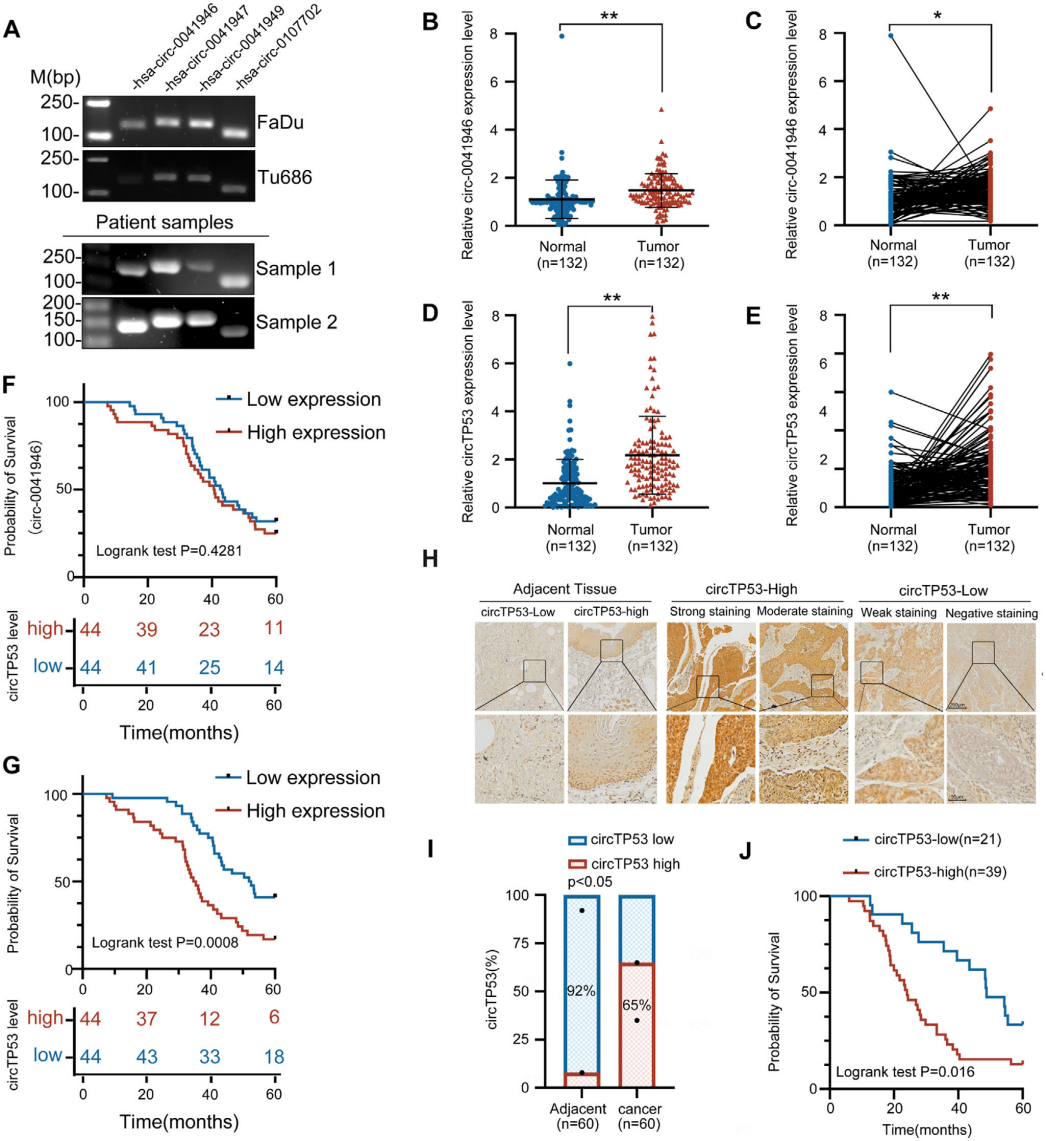
*hsa‐circ‐0041947* is a novel *circRNA* from the *TP53* gene associated with head and neck squamous cell carcinoma. A) The expression profiles of *circRNAs* derived from the TP53 gene in the normal human HNSCC cell lines (Up imaging) and the HNSCC tumors (Down imaging). B–E) The qRT‐PCR assay showing the relative levels of *hsa‐circ‐0041946* and *hsa‐circ‐0041947* (normalized to β‐actin) in the peritumor and tumor tissues of HNSCC (n = 132). F,G) Kaplan‐Meier analysis of correlations between *hsa‐circ‐0041946* and *hsa‐circ‐0041947* expression levels and OS (overall survival) of 88 HNSCC patients. H) Representative images of ISH staining of *circTP53* on TMAs. Scale bar, 200 µm. I) Quantification of *circTP53* expression in adjacent tissues (n = 60) and HNSCC tissues (n = 60). J) Overall survival (OS) curves of HNSCC patients with high or low *circTP53* levels. (Data are presented as the mean ± SEM, ^*^ *p* < 0.05, ^**^ *p* < 0.01).

To identify *circRNAs* originating from *TP53* and investigate their role in HNSCC development, we first analyzed the potential of *TP53* to generate *circRNAs* by examining online *circRNA* databases (circInteractome^[^ ^25^ ^]^ and circBase^[^ ^26^ ^]^). We identified five potential *circRNAs* originating from *TP53* (**Table** **1**). Utilizing the predicted sequences from these databases, we developed tailored divergent primers designed to selectively amplify the circular transcript to detect the presence of these *circRNAs* (Table S1, Supporting Information). The real‐time polymerase chain reaction (RT‐PCR) and agarose electrophoresis revealed that the 4 potential *circRNAs* were stably expressed in both HNSCC cell lines and HNSCC patient's tissues (**Figure** **1**A). To expound the expression pattern and clinical relevance of these *circRNAs* in HNSCC, quantitative real‐time polymerase chain reaction (qRT‐PCR) was employed to assess its expression level in a cohort of 90 paired clinical specimens of HNSCC and adjacent normal tissues. The results revealed a significant upregulation of *hsa_circ_00 41946* and *hsa_circ_00 41947* in tumor samples compared to adjacent normal tissue relative to other *circRNAs* derived from *TP53* (Figure 1B–E and Figure S1A–D, Supporting Information). Kaplan‐Meier analysis revealed that patients with higher *hsa_circ_00 41947* (furthermore called *circTP53* in this manuscript) expression experienced significantly shorter overall survival, while *hsa_circ_00 41946* showed no correlation with prognosis. (Figure 1F,G, Supporting Information).

**Table 1:** CircRNAs arised from TP53 as revealed by circBase and circlnteractome.

Circular RNA ID	Location	Genomic Length [bp]	Spliced length [bp]	RNAseq resources
hsa_circ_0041946	chr17:7573926‐7577608	3682	561	Salzman2013 (PMID:24039610)
hsa_circ_0041947	chr17:7573926‐7578554	4628	858	Salzman2013 (PMID:24039610)
hsa_circ_0041948	chr17:7576852‐7576926	74	74	Salzman2013 (PMID:24039610)
hsa_circ_0041949	chr17:7576852‐7577608	756	321	Rybak2015 (PMID:25921068) Salzman2013 (PMID:24039610)
hsa_circ_0107702	chr17:7576852‐7578554	1702	618	Rybak2015 (PMID:25921068)

circRNAs that are generated by back splicing of TP53 gene were analyzed with circBase and circlnteractome. Circular RNA ID, the location of circular RNA in the genome, genomic length, spliced length, and the RNAseq sources were shown

In this study, 192 patients were all squamous cell carcinomas (SCC) including SCC of the oral cavity, SCC of hypopharynx, and SCC of the larynx. *CircTP53* levels were higher in patients with lymph node metastases (N1 + N2) and stage III–IV disease, or distant metastases compared to patients with no lymph node involvement or stage I–II, or non‐metastatic cases. No significant correlation was observed in other clinicopathological features, including age, sex, tumor size, tumor site, and differentiation (**Table** **2**). To validate these findings, *circTP53* expression levels were also examined by immunohistochemistry staining in separate set of patients by HNSCC tissue array which contained 60 paraffin‐embedded HNSCC and 60 adjacent normal tissues. *CircTP53* was highly expressed in cancer tissue compared with adjacent normal tissue, with 65% of cancer samples classified as having a high expression rate (*circTP53*‐High) compared with only 8% of adjacent samples (Figure 1H,I). In this cohort, patients characterized by high *circTP53* expression also exhibited worse overall survival (OS) compared to patients with low *circTP53* expression. (Figure 1J). Together, these results suggest that high expression of *circTP53* correlates with worse prognosis in patients with HNSCC.

**Table 2:** The relationship between circTP53 expression and the clinicopathological characteristics of 192 HNSCC patients.

Characteristics		circTP53		Chi‐square	*p*‐Value
		Low	High		
Age	<50	29	26	0.23	0.63
	>50	67	70		
Gender	Male	75	83	2.29	0.13
	Female	21	13		
Tumor sites	Oral cavity	8	6	0.38	0.83
	Hypopharynx	58	61		
	Larynx	30	29		
Grade	I‐II	57	52	0.53	0.47
	III‐IV	39	44		
T stage	T1‐2	60	45	4.73	0.03^*^
	T3‐4	36	51		
N stage	NO	40	27	3.87	0.049^*^
	N1‐3	56	69		
TNM stage	I‐II	67	51	5.63	0.02^*^
	III‐IV	29	45		

^*^ *p* < 0.05

### 2.2 Characterization of *circTP53* in HNSCC

**Figure 2:**
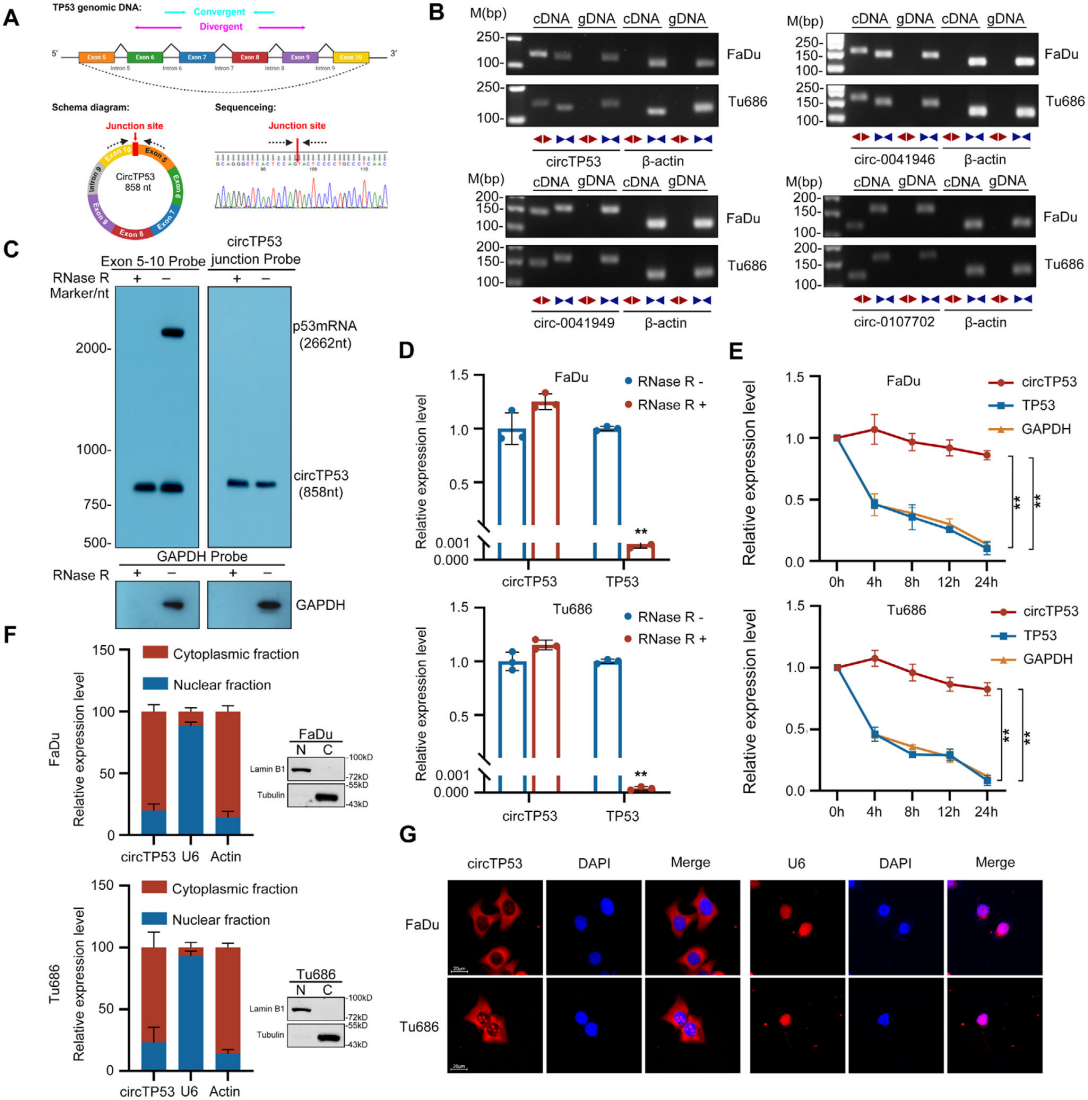
Analysis of characteristics of *circTP53* in HNSCC. A) Schematic illustration showing the *TP53* exon 5 exon 10 and intron 9(partial) circularization forming *circTP53*. The presence of *circTP53* was validated by RT‐PCR, followed by Sanger sequencing. Black arrow represents “head‐to‐tail” *circTP53* splicing sites. B) The presence of *circRNAs* derived from the *TP53* gene was validated in FaDu and Tu686 cell lines by RT‐PCR. Divergent primers amplified *circRNAs* in cDNA but not in genomic DNA. β‐actin was used as a negative control. C) Northern blotting analysis of *circTP53* and *TP53* mRNA levels in FaDu cells by hybridization with exon 10 (top, left) and exon 10‐exon 5 junction (top, right) probes with and without Rnase R treatment. GAPDH mRNA with or without RNase R treatment was detected as a control. D) qRT‐PCR analysis of the expression of *circTP53* and *TP53* mRNA after treatment with RNase R in FaDu and TU686 cells. E) qRT‐PCR for the abundance of *circTP53*, *TP53* mRNA and GAPDH mRNA in FaDu and Tu686 cells treated with Actinomycin D at the indicated time points. F) The levels of *circTP53* in the nuclear and cytoplasmic fractions of FaDu and Tu686 cells. Lamin B1 and Tubulin were detected as a protein control. G) FISH detection of *circTP53* in HNSCC cells. The nucleus was stained with DAPI. Scale bar, 20 µm. (Data are presented as the mean ± SEM, ns > 0.05, ^*^ *p* < 0.05, ^**^ *p* < 0.01) (All dots on the bar chart represent the mean value of each repeated experiment).

According to circbase, *circTP53* originates from exons 5 to 10 and contains partial intron 9 of *TP53*, spanning a length of 858 nucleotides. The back‐splicing site was validated through Sanger sequencing of RT–PCR products using specific divergent primers (**Figure** **2**A). To rule out the possibility that the head‐to‐tail splicing of *circTP53* was produced by genomic rearrangements or PCR artifacts, RT–PCR was performed using convergent and divergent primers (Table S1, Supporting Information), respectively. The gel electrophoresis of RT‐PCR products revealed that 4 potential *circRNAs* were amplified by divergent primers in cDNA, but not in gDNA (Figure 2B). Moreover, we used probes that hybridize with the splicing junction to distinguish *circTP53* and probes that hybridize with exon5‐10 to distinguish *circTP53* and its mRNA by northern blotting and used GAPDH mRNA primers as a control. This confirmed that *circTP53* was about 800 nt, consistent with the *hsa_circ_00 41947* annotation (Figure 2C). *CircTP53* was resistant to the degradation by RNase R, while *TP53* mRNA was degraded (Figure 2D). To further validate that *circTP53* harbors a stable cyclic structure, we treated FaDu and Tu686 cells with Actinomycin D and found that *circTP53* was much more stable than *TP53* mRNA (Figure 2E). Further analysis through nuclear and cytoplasmic fractionation revealed the presence of *circTP53* in both compartments, with a predominant localization in the cytoplasm of HNSCC cells (Figure 2F). This localization pattern was corroborated by the fluorescence in situ hybridization assay (FISH), where U6 RNA was used as the nuclear marker (Figure 2G). These results collectively reveal that *circTP53* is an abundant and stable *circRNA* expressed in the cytoplasm of HNSCC cells that are generated from *TP53* by back splicing.

### 2.3 *CircTP53* Promotes HNSCC Progression by Promoting Cell Proliferation, Migration, Invasion, and Inhibiting Cell Apoptosis

**Figure 3:**
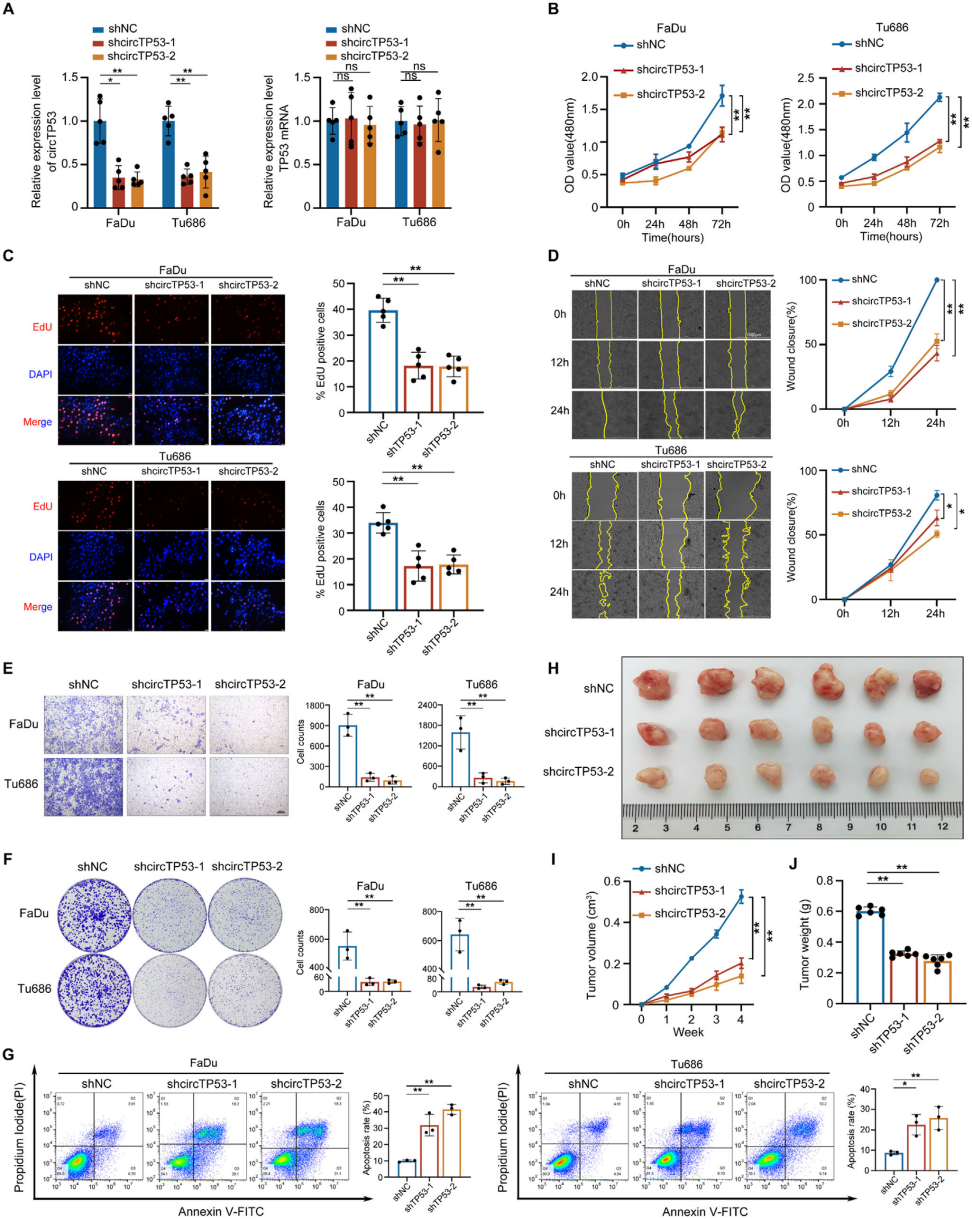
*circTP53* promotes HNSCC progression by promoting cell proliferation, migration, invasion, and inhibiting apoptosis. A) The shNC was used as the negative control and sh*circTP53*‐1 and sh*circTP53*‐2 were used as stable *circTP53* knockdown cell lines. The qRT‐PCR analysis showed that *shcircTP53‐1*/*shcircTP53‐2* significantly reduced *circTP53* expression in HNSCC cell lines (*p* < 0.05), while TP53 mRNA levels remained unchanged. B) CCK‐8 were conducted in HNSCC cells after *circTP53* depletion. C) DNA synthesis assessed using a 5‐ethynyl‐20‐deoxyuridine (EdU) assay in indicated cells (left panel). Scale bar: 100 mm. Quantitative data of EdU assay (right panel). D) Wound healing assay was used to detect the migration of *circTP53* depletion HNSCC cells (left panel). The analysis of relative wound closure rate (right panel). E) The invasion ability of *circTP53*‐depleted HNCC cells as detected by transwell assays (left panel). The analysis of relative cell counts (right panel). F) Colony formation assay of *circTP53*‐depleted HNCC cells (left panel). The analysis of relative colony formation rate (right panel). G) apoptosis rate of HNSCC cells transfected with *circTP53*‐depleted were analyzed by flow cytometry. H) Stable *circTP5*3 knockdown (shcircTP53‐1/shcircTP53‐2) or negative control (shNC) FaDu cell lines were used to establish a xenograft tumor model in nude mice. Photograph of xenograft tumors removed from each nude mouse (n = 6). I) Growth curves of xenograft tumors of each group of nude mice were minored and measured once a week. J) Tumor weight was calculated. (Data are presented as the mean ± SEM, ns > 0.05, ^*^ *p* < 0.05, ^**^ *p* < 0.01) (All dots on the bar chart represent the mean values of each repeated experiment).

To study the possible function of *circTP53* in HNSCC, we generated *circTP53* stable knockdown (shcircTP53‐1 and shcircTP53‐2) and overexpression (OE circTP53) cell lines in both parent FaDu and Tu686 cells. QRT‐PCR and fluorescence verification of GFP tags were performed to confirm that the overexpression or knockdown efficiency of *circTP53* in these cell lines. The qRT‐PCR analysis showed that both FaDu and Tu686 transfected shcircTP53‐1 and shcircTP53‐2 had significantly 2.8‐4‐fold reduced *circTP53* expression (all *p* < 0.05), while these cells with OE circTP53 had significantly 3‐4‐fold increased *circTP53* expression (all *p* < 0.05) without TP53 mRNA level change (**Figures** **3**A and S2A–C, Supporting Information). Cell proliferation assay, wound healing assay, and transwell migration assay were performed with Matrigel‐coated chambers to assess the effect of *circTP53* on cell proliferation, migration, and invasion. CCK‐8 growth curves and EdU assay indicated that the knockdown of *circTP53* in FaDu and Tu686 cells led to significant inhibition of cell viability (Figure 3B,C). Next, the wound healing assay showed that the knockdown of *circTP53* significantly impaired the migration of HNSCC cell lines (Figure 3D). Knockdown of *circTP53* dramatically decreased the invasion of HNSCC cells (Figure 3E). By clonogenic assay, the knockdown of *circTP53* lowered both the number of clones and the clonal proliferative capacity of HNSCC cells (Figure 3F). Furthermore, the knockdown of *circTP53* resulted in an increased apoptosis rate of FaDu and Tu686 cells (Figure 3G). To investigate the effect of *circTP53* on HNSCC cell growth in *vivo*, FaDu cell lines with stable *circTP53* knockdown were inoculated into the four‐week‐old female BALB/c‐nude mice to establish HNSCC xenograft models. After 4 weeks, the knockdown of *circTP53* significantly reduced tumor growth in *vivo* (Figure 3H‐J). Collectively, these data demonstrated that *circTP53* promoted proliferation and migration, and inhibited apoptosis in HNSCC cells.

### 2.4 *circTP53* Interacts with usp10 and Stabilizes Mutually

To explore the potential role of *circTP53* in HNSCC, we analyzed the sequence information of *circTP53* using the online database *circRNA*Db (http://reprod.njmu.edu.cn/cgi‐bin/circRNAdb/circRNADb.php) and found two potential IRES elements and one potential open reading frame (ORF) (Figure S3A,B, Supporting Information). To facilitate the detection of potential protein expression, we inserted a nucleotide sequence encoding a Flag tag upstream of the stop codon (TAA) within the potential ORF in the pLC5‐ciR‐*circTP53* plasmid (Figure S3C, Supporting Information). To prevent false‐positive results caused by linear transcripts, we introduced a frameshift mutation at the RNA circularization site in the aforementioned plasmid, ensuring that the plasmid expressed only the circular RNA while the linear transcript could not be translated due to the absence of a start codon upstream of the Flag sequence (Figure S3D, Supporting Information). Western blot results indicate that the overexpression plasmid can express the Flag tag, while the overexpression plasmid with the modified junction site could not (Figure S3E, Supporting Information). This suggests that *circTP53* may not be translated into protein.

**Figure 4:**
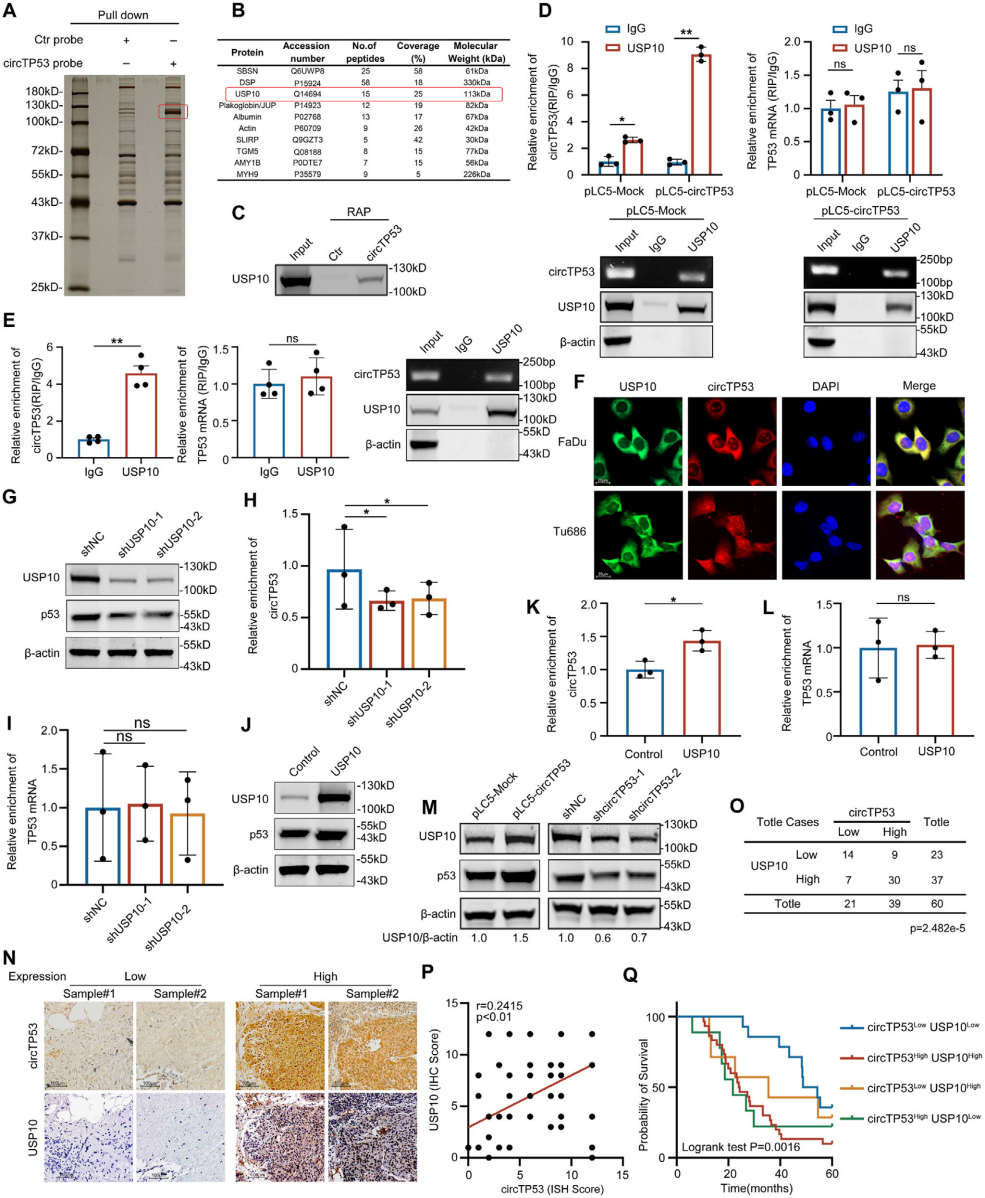
*circTP53* and usp10 interact and stabilize each other. A) Silver staining of proteins pulled down by biotin‐labeled probe specific for *circTP53* and control probe. B) The RNA pull‐down proteins identified by mass spectrometry analysis. C) The interaction between *circTP53* and USP10 was verified by RAP assay and WB. D,E) RIP assays of endogenous and exogenous were carried out in FaDu cells under indicated conditions using anti‐USP10 and IgG control, followed by qRT‐PCR of *circTP53* and *TP53* mRNA. F) FISH and IF co‐staining indicating the co‐localization of *circTP53* (red) and USP10 (green) in HNSCC cells. Nuclear were stained with DAPI. Scale bar, 20 µm. G–I) Western blot analyses of FaDu cells after knockdown of USP10 with indicated antibodies, followed by qRT‐PCR of *circTP53* (H) and *TP53* mRNA (I). J–L) Western blot analyses of FaDu cells after overexpression of USP10 with indicated antibodies, followed by qRT‐PCR of *circTP53* (K) and *TP53* mRNA (L). M) Western blot analyses of FaDu cells after knockdown of *circTP53* with indicated antibodies. N–P) USP10 protein levels show a positive correlation with expression of *circTP53* in HNSCC tumor tissues (N), and statistical analysis of all tumor samples was shown in (O) (calculated by Pearson's chi‐squared test) and (P) (calculated by Pearson's correlation). (Scale bars, 100 µm). Q) High expression of *circTP53* and high expression of USP10 are correlated with the lowest overall survival rate. Kaplan‐Meier analysis was performed in four groups of HNSCC tumor tissues, with log‐rank test, *p* = 0.0016. (Data are presented as the mean ± SEM, #*p* > 0.05, ^*^ *p* < 0.05, ^**^ *p* < 0.01) (All dots on the bar chart represent the mean values of each repeated experiment).

Thus, we focus our attention on the function of *circRNA* binding with proteins. RNA pull‐down assay was performed with a biotin‐labeled probe targeting the back‐splicing site of *circTP53* in FaDu cells, followed by mass spectrometry (**Figure** **4**A). Notably, *circTP53* was found to pull down USP10, and its proteins and peptides were identified through mass spectrometry (Figure 4B and Figure S4A, Supporting Information). To confirm this finding, the RAP assay and western blotting were used to verify the interaction between *circTP53* and USP10 in HNSCC FaDu and Tu686 cell lines as well as patients’ adjacent and tumor tissues (Figure 4C; Figure S4B,C, Supporting Information). The exogenous and endogenous *circTP53* interaction with USP10 was further confirmed through RIP assay in both the same HNSCC cell lines and patient's adjacent and tumor tissues. Moreover, the qRT‐PCR analysis revealed no significant correlation between the mRNA linear transcript of *TP53* and USP10 (Figure 4D,E and Figure 4D–K, Supporting Information). Immunofluorescence analysis further supported the interaction, indicating colocalization of *circTP53* with USP10 within the cytoplasm (Figure 4F).

To elucidate the reciprocal impact of the interaction between *circTP53* and USP10 on their individual stability, we generated FaDu cell lines with USP10 knocked down or overexpressed to evaluate *circTP53* and *TP53* mRNA expression levels using qRT‐PCR. Knockdown or overexpression of USP10 significantly reduced or increased the levels of *circTP53* and p53 protein but does not affect the levels of p53 mRNA (Figure 4G–L, Supporting Information). Concurrently, we observed that knockdown and overexpression of *circTP53* similarly decreased or increased the protein levels of USP10 and p53, without affecting the mRNA levels of USP10 (Figure 4M). We observed a significant correlation between low or high expression level of *circTP53* and USP10 protein level (Figure 4N). In addition, we found that the correlation between protein levels of USP10 and the expression levels of *circTP53* was significant (Figure 4O,P). The overall survival rate of the patients with high expression levels of *circTP53* and high expression of USP10 was much lower than that of the patients with low expression levels of *circTP53* and low USP10 expression (Figure 4Q). Taken together, this data supports the role of a direct interaction between *circTP53* usp10, which stabilizes each other.

### 2.5 Intron 9 in *circTP53* Interacts with 100–399 AA of USP10

**Figure 5:**
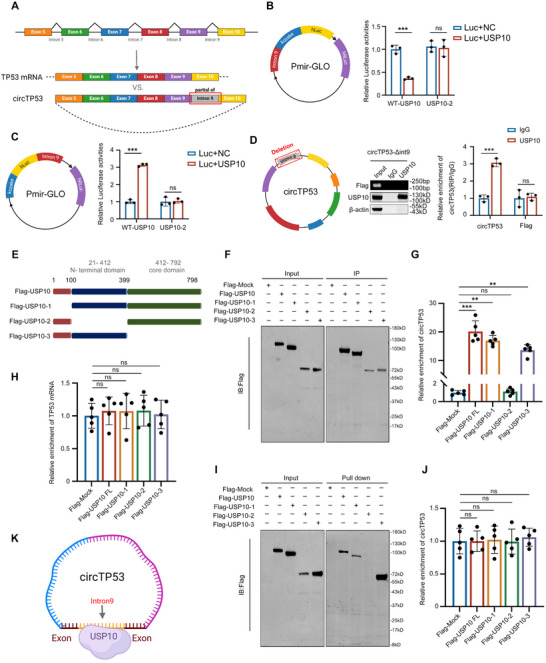
*circTP53* segment transcribed from partial intron 9 interacts with 100–399 AA of USP10. A) Schematic diagram comparing *circTP53* sequences to p53 mRNA obtained from NCBI. B,C) Schematic illustration displayed the dual‐luciferase report vectors with intron 9 (partial) binding sites. And the relative activities of luciferase were detected after co‐transfection of USP10‐WT, USP10‐MT and negative control respectively in FaDu cells. D) RIP assays of exogenous were carried out in FaDu cells after delete intron 9 using anti‐USP10 and IgG control, followed by qRT‐PCR of *circTP53*. E) Schematic diagram of full‐length and truncated USP10 protein. F–H) RIP assays were executed with anti‐Flag in FaDu cells transfected with indicated full‐length or truncated USP10 plasmids with Flag tags. Co‐precipitated proteins and RNAs were purified and followed by western blot and qRT‐PCR, respectively. I,J) RNA pull‐down assays using biotin‐labeled *circTP53* probe in FaDu cells expressing full‐length of USP10 and its deletion mutants. The pulled‐down proteins were subjected to western blot and followed by qRT‐PCR of *circTP53*. K) Schematic diagram illustrating the generation of intron 9 (partial) of *circTP53* interacts with USP10. (Data are presented as the mean ± SEM, ns > 0.05, ^*^ *p* < 0.05, ^**^ *p* < 0.01) (All dots on the bar chart represent the mean values of each repeated experiment).

To elucidate how USP10 affects *circTP53* expression without influencing the linear transcript mRNA, we conducted sequence alignment of *circTP53*, p53 mRNA, and other circular RNAs derived from *TP53*. As shown in (**Figure** **5**A), partial sequence of intron9, suspected to be involved in binding with USP10, is contained in *circTP53* sequences more abundantly compared to p53 mRNA. To functionally study this, dual‐luciferase reporter assays were constructed, where Intron9 (partial) was separately cloned into the promoter region and 3′UTR of the reporter plasmid. For this purpose, dual‐luciferase reporter assays were conducted, where Intron9 (partial) was separately cloned into the promoter region and 3′UTR of the reporter plasmid. The results indicated that USP10 significantly decreased the luciferase activity of cells transfected with the reporter plasmid containing Intron9 (partial) inserted into the promoter region (Figure 5B), while enhancing the luciferase activities of cells transfected with the reporter plasmid containing Intron9 (partial) inserted into the 3′UTR region (Figure 5C). Subsequently, mutant plasmids carrying Flag‐tagged with exogenous deletion of Intron9 (partial) were used for RIP experiments, revealing that the deletion of Intron9 (partial) resulted in the loss of *circRNA*’s ability to interact with USP10. (Figure 5D). As shown in Supplementary Figure S5A, *hsa_circ_00 41946* also contains Intron9 (partial), leading us to speculate that *hsa_circ_00 41946* may also interact with USP10. The exogenous *hsa_circ_00 41946* interaction with USP10 was additionally confirmed through RIP assay (Supplementary Figure S5B). And knockdown or overexpression of USP10 reduced or increased the levels of *hsa_circ_00 41946* and p53 protein but did not affect the levels of p53 mRNA (Figure S5C–H, Supporting Information). These findings indicated that the transcribed portion of intron 9 in *circTP53* was the sequence that interacted with USP10 protein.

To identify the amino acid sequence in USP10 that interacts with *circTP53*, we constructed Flag‐tagged full‐length USP10 and its truncation mutants, aligning with the functional domains of USP10 (Figure 5E). RIP assay revealed that the N‐terminal region (100–399 aa) of USP10, rather than other domains, played a crucial role in its interaction with *circTP53*, while the linear form of mRNA for *TP53* remained unbound (Figure 5F–H). Furthermore, RNA pull‐down assay detected the interaction between endogenous *circTP53* and the N‐terminal region (100–399aa) of USP10 (Figure 5I,J). Importantly, it was the wild‐type USP10, not the 100–399aa deletion variant (USP10‐2), that significantly affected luciferase activity in the dual‐luciferase assays (Figure 5B,C). Therefore, 100–399 AA of USP10 interacts with *circTP53* segment from partial intron 9 (Figure 5K).

### 2.6 USP10 Promotes Tumor Progression in HNSCC

**Figure 6:**
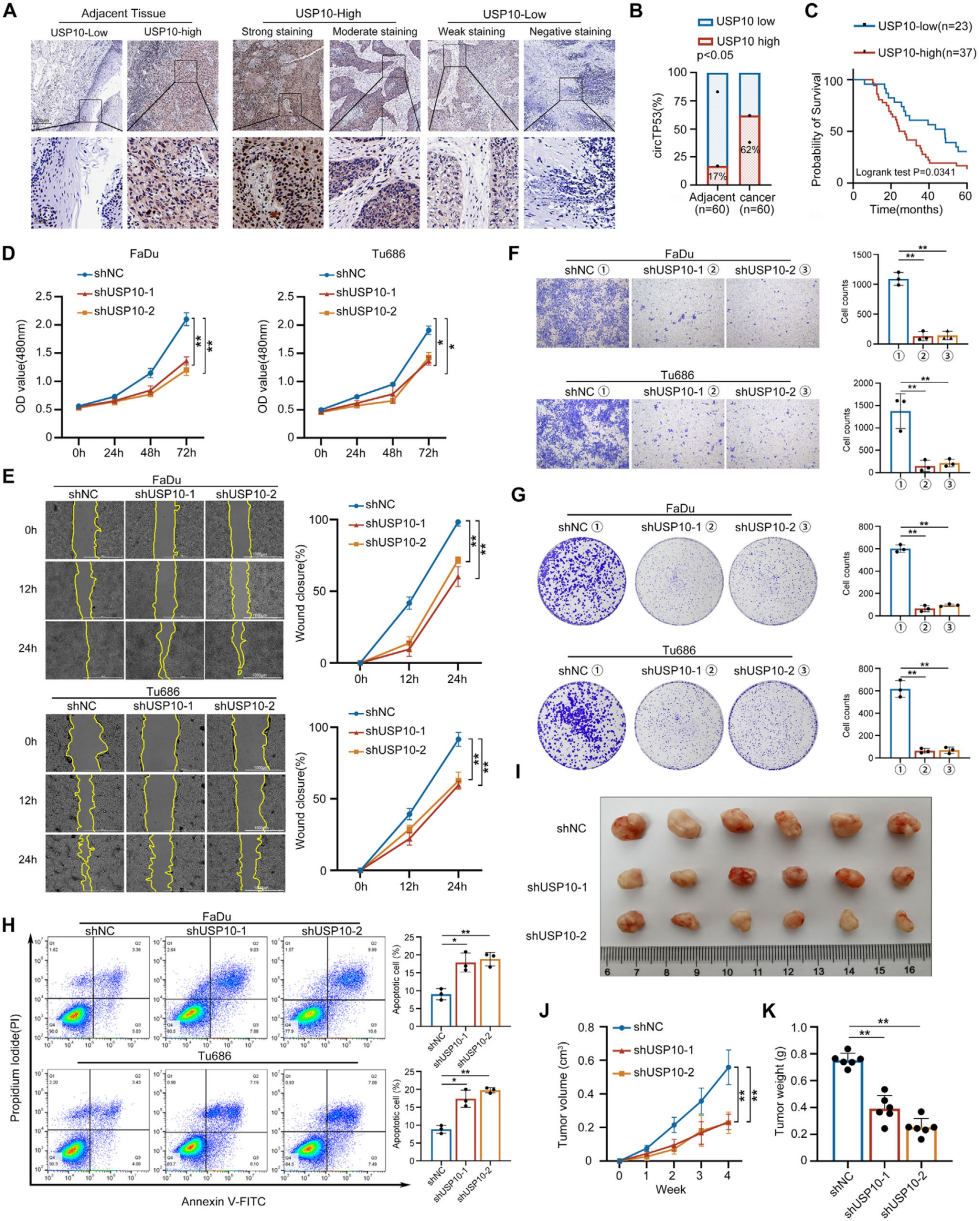
USP10 promotes tumor progression in HNSCC. A) Immunohistochemistry (IHC) staining of USP10 in adjacent tissues and HNSCC tissues. Representative images are shown (Upper, 40×; Lower, 400×). B) Quantification of USP10 expression in adjacent tissues (n = 60) and HNSCC tissues (n = 60). C) Overall survival (OS) curves of HNSCC patients with high or low USP10 levels. D) shNC as the negative control and shUSP10‐1/shUSP10‐2 as the stable USP10 knockdown cell lines. CCK‐8 were conducted in HNSCC cells after USP10 depletion. E) Wound healing assay was used to detect the migration of USP10 depletion HNSCC cells (left panel). The analysis of relative wound closure rate (right panel). F) The invasion ability of USP10‐depleted HNCC cells as detected by transwell assays (left panel). The analysis of relative cell counts (right panel). G) Colony formation assay of USP10‐depleted HNCC cells (left panel). The analysis of relative colony formation rate (right panel). H) Apoptosis rate of HNSCC cells transfected with USP10‐depleted were analyzed by flow cytometry. I) The shUSP10‐1/shUSP10‐2 and shNC FaDu cell lines were used to establish a xenograft tumor model in nude mice. Photograph of xenograft tumors removed from each nude mouse (n = 6). J) Growth curves of xenograft tumors of each group of nude mice were minored and measured once a week. K) Tumor weight was calculated. (Data are presented as the mean ± SEM, ns > 0.05, ^*^ *p* < 0.05, ^**^ *p* < 0.01) (All dots on the bar chart represent the mean values of each repeated experiment).

To investigate the clinical relevance of USP10 in patients with HNSCC, IHC staining was conducted on HNSCC and adjacent normal tissue. USP10 exhibited significantly higher expression levels in HNSCC tissue compared to adjacent tissues, with 62% of HNSCC samples classified as having a high expression rate (USP10‐High), in contrast to only 17% of adjacent samples (**Figure** **6**A,B). Patients with USP10‐High showed significantly shorter OS than patients with low USP10 expression (Figure 6C). Cell proliferation, wound healing, and Transwell assays were conducted to assess the effect of USP10 on cell proliferation and migration. CCK‐8 growth curves indicated that knockdown of USP10 in HNSCC cell lines led to significant inhibition of cell viability (Figure 6D). The wound healing assay showed that the knockdown of USP10 significantly impaired the migration of HNSCC cell lines (Figure 6E) and dramatically decreased the invasion of HNSCC cells (Figure 6F). By clonogenic assay, knockdown of USP10 lowered both the number of clones and clonal proliferative capacity of HNSCC cells (Figure 6G). Furthermore, knockdown of USP10 resulted in an increased apoptosis rate of HNSCC cells (Figure 6H). Collectively, these data demonstrated that USP10 promoted proliferation and migration, and inhibited apoptosis of HNSCC cells. To investigate the effect of USP10 on HNSCC growth in *vivo*, FaDu cells with USP10 stable knockdown were subcutaneously inoculated into the axillae of four‐week‐old female BALB/c‐nude mice to establish HNSCC xenograft models. Four weeks later, we found that loss expression of USP10 significantly retarded the tumor growth in *vivo* (Figure 6I–K). Analyzing the relationship between the differential expression of USP10 mRNA and prognosis using the GAPIA2 website revealed that patients with higher USP10 expression experienced significantly shorter OS by Kaplan‐Meier analysis. (Figure S6, Supporting Information). Together, these data indicated that USP10 regulates HNSCC progression.

### 2.7 *circTP53* Stabilizes p53 by Stabilizing usp10 and Promoting Deubiquitination of p53 by usp10

As reported, in lung cancer and breast cancer, USP10, an upstream deubiquitinase of p53, prevents the targeted proteasomal degradation of p53 by removing its polyubiquitination.^[^ ^23^, ^27^ ^]^ To verify the interaction between USP10 and p53 in HNSCC, Co‐IP assays were conducted in FaDu cells, revealing the coimmunoprecipitation of USP10 and p53 (Supplementary Figure S7A). Deletion‐mapping investigations using Flag‐tagged full‐length USP10, and its truncated mutants unveiled that the N‐terminal region (1‐100aa) of USP10 played a crucial role in the interaction with p53 (Supplementary Figure S7B). After this, we examined the impact of USP10 on p53 ubiquitination in FaDu cells. Our findings indicate a reduction in p53 ubiquitination upon upregulation of USP10 (Figure S7C, Supporting Information). Furthermore, the knockdown of USP10 resulted in a significant decrease in the protein levels of p53 and its downstream targets, p21 and Bax (Figure S7D, Supporting Information). Together, these findings demonstrate that USP10 functions to deubiquitinate and stabilize p53 in HNSCC.

**Figure 7:**
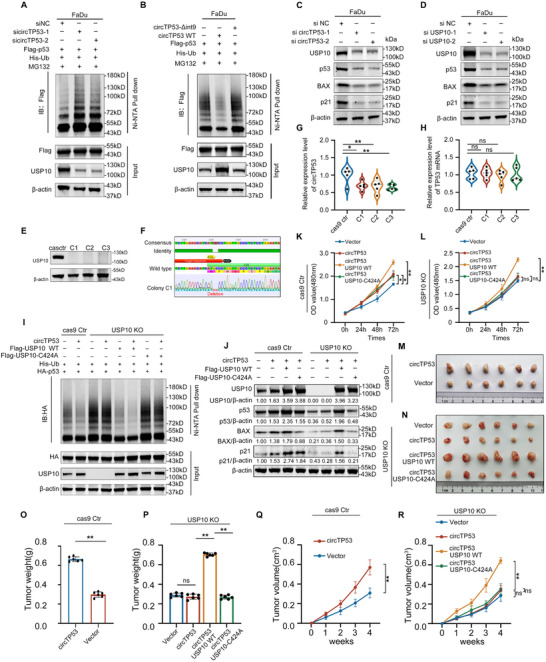
*circTP53* stabilizes p53 by stabilizing usp10 and promoting deubiquitination of p53 by usp10. A) Ubiquitination levels of p53 were detected in MG132‐treated FaDu cells co‐transfected with indicated vectors or siRNAs. B) Ubiquitination levels of p53 were detected in MG132‐treated FaDu cells co‐transfected with *circTP53* WT or *circTP53* MT. C) Western blot analyses of FaDu cells after knockdown of *circTP53* with indicated antibodies. D) Western blot analyses of FaDu cells after knockdown of USP10 with indicated antibodies. E) immunoblot analysis of WT and KO:FaDu cells KO for USP10 using three sgRNAs (C1, C2, C3). β‐actin serves as a loading control. F) Schematic diagram of sequence alignment of KO sequencing. G,H) QRT‐PCR of *circTP53* (G) and *TP53* mRNA (H) after knockout of USP10. I) Western blot analysis for ubiquitination levels of p53 showing the rescue of KO phenotype by *circTP53*. Here, we show FaDu cells expressing WT levels of USP10, KO USP10 (C1), or complemented USP10 (KO+ overexpressed USP10 WT and USP10‐C424A). β‐actin serves as a loading control. J) Western blot analyses of FaDu cells after KO USP10 showing the rescue of KO phenotype by *circTP53* with indicated antibodies. K,L) CCK‐8 were conducted in cas9 control cells and USP10 KO cells after transfecting USP10 WT or USP10‐C424A. M,N) Photograph of xenograft tumors removed from each nude mouse after 4 weeks (n = 6). O,P) Growth curves of xenograft tumors of each group of nude mice were minored and measured once a week. Q,R) Tumor weight was calculated. (Data are presented as the mean ± SEM, ns > 0.05, ^*^ *p* < 0.05, ^**^ *p* < 0.01) (All dots on the bar chart represent the mean values of each repeated experiment).

As *circTP53* facilitates the stabilization of p53 protein (Figures 4M and 3A), we hypothesized that the stabilization of p53 protein by *circTP53* is mediated through USP10‐mediated deubiquitination of p53. Indeed, ubiquitination assay experiments showed that knockdown of *circTP53* enhanced p53 poly‐ubiquitination, while overexpression of *circTP53*, but not *circTP53*‐Δint9, could lead to a reduction in poly‐ubiquitination of p53 in FaDu cells (**Figure** **7**A,B). Importantly, western blot revealed that knockdown of *circTP53*, like knocking down USP10, inhibited the protein level of p53 as well as p21 and Bax in HNSCC cells (Figure 7C,D). To further determine the impact of *circTP53* on the levels of p53 through its stabilization of USP10, we deleted USP10 using CRISPR‐Cas9 in HNSCC cell lines using three sgRNAs (Figure 7E), and a sequence comparison was performed (Figure 7F). The qRT‐PCR results indicated a significant reduction in the content of *circTP53*, while the mRNA of parent gene content remained unchanged (Figure 7G,H). Notably, in the Cas9 control cell line, overexpression of *circTP53* resulted in reduced ubiquitination and increased levels of p53, whereas in the USP10 knockout (USP10 KO cell line), overexpression of *circTP53* did not significantly alter the polyubiquitination or levels of p53. Subsequently, we conducted rescue experiments by introducing USP10‐WT and USP10‐MT (C424A). Our results indicated that in the USP10 KO cell line, samples with overexpression of both *circTP53* and USP10‐WT exhibited significantly reduced polyubiquitination and increased levels of p53 (Figure 7I,J). This indicates that *circTP53*‐mediated deubiquitination of p53 is dependent on USP10. At the cellular level, the CCK8 cell proliferation assay also demonstrated that in the KO cell line, introducing USP10 WT, rather than USP10‐C424A, rescued the deubiquitination of p53 mediated by *circTP53* (Figure 7K,L).

To further validate the previously mentioned results, we investigated the reciprocal effect between *circTP53* and USP10 in *vivo*. After 4 weeks, overexpression of *circTP53* in control cell lines significantly promotes tumor progression, whereas in USP10 knockout cells, overexpression of *circTP53* showed no significant difference compared to the control group. Importantly, this result can be rescued by overexpression of USP10 WT rather than mutant USP10 (C424A)^[^ ^28^ ^]^ (Figure 7M–R). These results suggested that *circTP53* stabilizes p53 by stabilizing usp10 and promoting deubiquitination of p53 by usp10, thereby regulating the progression of HNSCC.

### 2.8 Effect of *circTP53* on Tumor Progression Depends on the Mutation Status of p53 Gene

As a crucial tumor suppressor, wtp53 exerts anti‐cancer effects, whereas most mutant forms of p53 promote tumor progression through other mechanisms, such as gain of function (GOF), loss of function (LOF), dominant negative effect (DNE).^[^ ^29^, ^30^ ^]^ Consequently, *circTP53* and USP10 may exert dual regulatory effects on tumor progression in HNSCC. However, our functional results paradoxically show that both *circTP53* and USP10 significantly promote HNSCC progression at both cellular and tissue levels.

**Figure 8:**
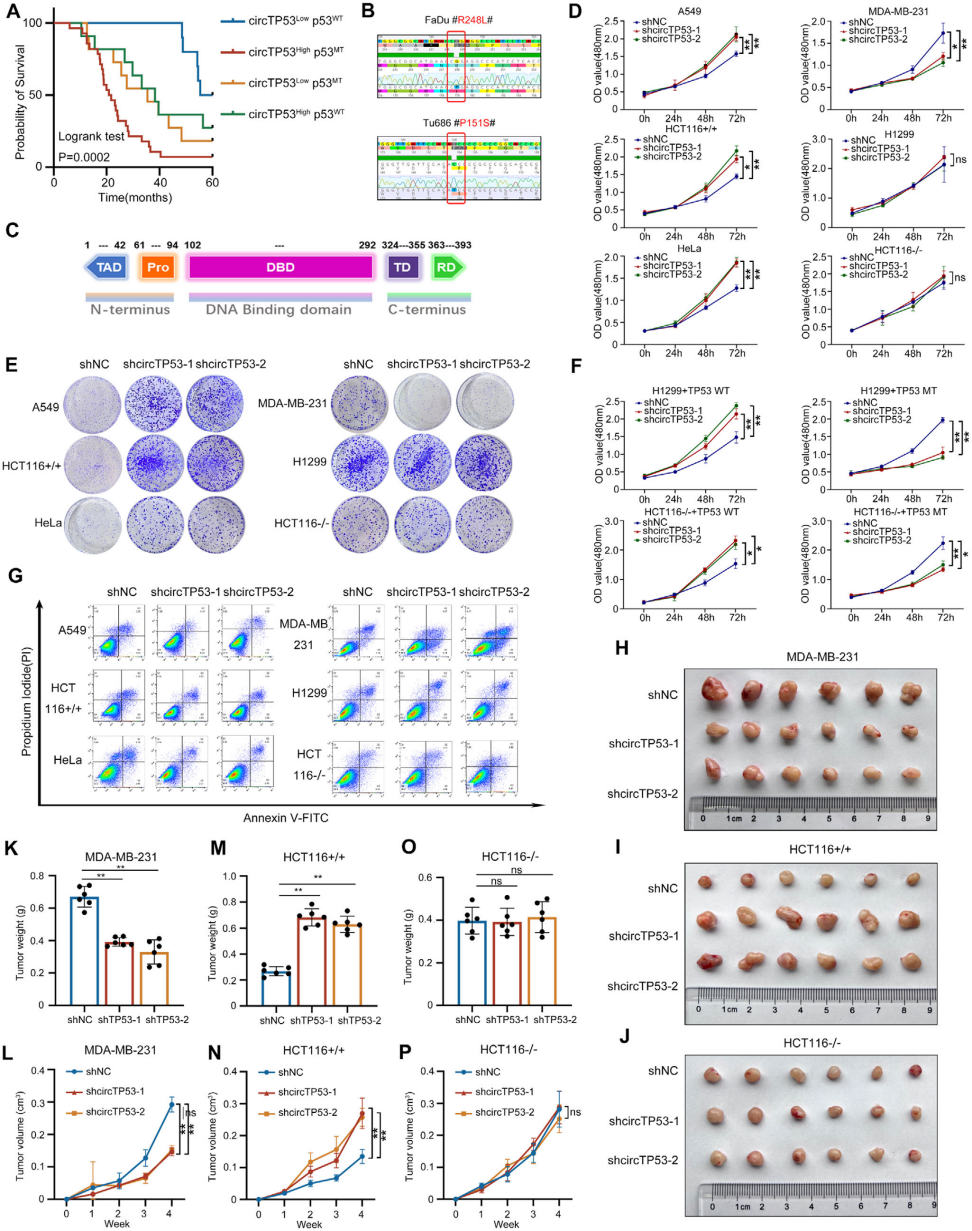
The regulation of *circTP53* on tumor progression depends on the mutation status of p53 gene. A) High expression of *circTP53* and p53 MT are correlated with the lowest overall survival rate. Kaplan‐Meier analysis was performed in four groups of HNSCC tumor tissues, with log‐rank test, *p* = 0.0002. B) Sequence alignment of Mutation sites of p53 gene sequencing in HNSCC cells. C) The functional domains in the p53 proteins. (AD, activation domain; PRD, proline‐rich domain; DBD, DNA‐binding domain; NLS, nuclear localization signal; TD, tetramerization domain; BD, basic domain). D) CCK‐8 were conducted in pan‐cancer cells after *circTP53* depletion. E) Colony formation assay of *circTP53*‐depleted pan‐cancer cells. F) CCK‐8 were conducted in pan‐cancer cells after transfection of USP10‐WT or USP10‐MT. G) Apoptosis rate of pan‐cancer cells transfected with *circTP53*‐depleted were analyzed by flow cytometry. H–J) Photograph of xenograft tumors removed from each nude mouse (n = 6). K,M,O) Tumor weight was calculated. L,N,P) Growth curves of xenograft tumors of each group of nude mice were minored and measured once a week. (Data are presented as the mean ± SEM, ns > 0.05, ^*^ *p* < 0.05, ^**^ *p* < 0.01) (All dots on the bar chart represent the mean values of each repeated experiment).

Upon analysis, we hypothesized that the significant oncogenic effects of *circTP53* and USP10 may be due to the high frequency of missense mutations in p53 in HNSCC. Hence, we regrouped tissue samples based on the mutant status of p53. We found that patients with mtp53 and high *circTP53* expression had poor prognoses. Similarly, patients with high USP10 expression and mtp53 also exhibited poor prognoses (**Figures** **8**A and S8A, Supporting Information). We also performed sequencing identification on cell lines and found that FaDu and Tu686 harbor mutations at 248 and 151 sites in the DNA‐binding domain (DBD) of the *TP53* gene, respectively (Figure 8B,C). According to previous reports, these mutations are associated with GOF effects.^[^ ^31^, ^32^ ^]^

To further determine the role played by *circTP53* in the diverse states of p53 in tumors, we introduced pan‐cancer cell lines (MDA‐MB‐231, A549, H1299, HCT116 p53^−/−^, HCT116 p53^+/+^ and Hela) (Figure 8B, Supporting Information) and assessed their epigenetic effects in tumors after knocking down and restoring *circTP53*. In p53 WT cell lines (A549, HCT116 p53^+/+^, HeLa), knockdown of *circTP53* promoted cell viability. Conversely, in the p53 MT cell line (MDA‐MB‐231), *circTP53* knockdown inhibits cell viability, while in p53 null cell lines (H1299, HCT116 p53^−/−^), *circTP53* knockdown has no significant effect on cell viability (Figure 8D,E). Notably, restoring mtp53 expression in H1299 and HCT116 p53^−/−^ cells results in increased cell viability upon *circTP53* depletion, whereas restoring the mtp53 (R248L) instead wtp53 in these cells leads to decreased cell viability following *circTP53* knockdown (Figure 8F). In apoptosis assays, *circTP53* depletion inhibits apoptosis in A549, HCT116 p53^+/+^, and HeLa cells, promotes apoptosis in MDA‐MB‐231 cells, and shows no significant effect in H1299 and HCT116 p53^−/−^ cells (Figure 8G). Similarly, in vitro xenograft experiments show that stable knockdown of *circTP53* significantly suppresses tumor growth in MDA‐MB‐231 cells, promotes tumor growth in HCT116 p53^+/+^ cells, and has no significant effect on tumor growth in HCT116 p53^−/−^ cells (Figure 8H‐P). These results suggested that the regulation of *circTP53* on tumor progression depends on the mutation status of p53 gene.

## 3 Discussion

In this study, we identified a significant correlation between *circTP53* expression and HNSCC clinical outcomes. Notably, high expression of *circTP53* exhibited in HNSCC tumor tissues is indicative of poor prognosis. Mechanistically, *circTP53* interacts with the upstream deubiquitinase USP10 of the p53 protein, resulting in mutual stabilization. Through its interaction with the amino acids 100–399 of USP10 via the partial intron 9 sequence derived from the parental gene, *circTP53* enhances USP10 stability, thereby augmenting its deubiquitination effect on p53 and reducing p53 degradation by the proteasome, consequently elevating p53 protein levels. Importantly, the effects of *circTP53* on p53 ubiquitination, downstream gene expression, cell viability, and in *vitro* xenograft tumor growth are dependent on USP10. These findings highlight the *circTP53*/USP10/p53 axis as a potential therapeutic target for HNSCC.

As the seventh most common cancer worldwide, HNSCC often manifests insidiously, with many patients diagnosed at advanced stages.^[^ ^2^ ^]^ Current treatment predominantly relies on traditional approaches such as surgery, radiotherapy and chemoradiotherapy due to the lack of effective targeted therapies.^[^ ^1^ ^]^ Recent research has highlighted the close association between *circRNAs* and tumor development, with an increasing diversity of types and quantities being discovered.^[^ ^33^, ^34^ ^]^ For instance, *circPTEN* promotes metastasis by disrupting the Smad4‐Smad2/3 interaction and suppressing downstream genes linked to epithelial‐mesenchymal transition,^[^ ^35^ ^]^ and *circEGFR* as an autophagy‐responsive *circRNA* implicated in Triple‐negative breast cancer (TNBC) progression and metastasis, suggesting its potential as a diagnostic biomarker and therapeutic target for TNBC.^[^ ^36^ ^]^

This study primarily investigated the generation of four *circRNAs* by *circTP53*, labeled as *hsa_circ_00 41946*, *hsa_circ_00 41947*, *hsa_circ_00 41949*, and *hsa_circ_010 7702*, respectively, with the *hsa_circ_00 41948* not further explored due to its shorter nucleotide sequence. Among these four *circRNAs*, only *circRNAs hsa_circ_00 41946* and *hsa_circ_00 41947* were found to be highly expressed in tumors, with further exploration indicating that only *hsa_circ_00 41947* was associated with patient survival staging and prognosis. Mechanistically, we discovered that both *circRNAs hsa_circ_00 41946* and *hsa_circ_00 41947* contained sequence segments derived from intron 9 (partial) and could interact with USP10. However, only *hsa_circ_00 41947* could interact with USP10 and mutually stabilize each other, whereas USP10 could stabilize *hsa_circ_00 41946*, but *hsa_circ_00 41946* could not increase the levels of USP10. Comparative sequence analysis indicated that *hsa_circ_00 41946* lacked specific sequences, suggesting the possible interaction of other proteins with *hsa_circ_00 41947* but not *hsa_circ_00 41946*, thereby potentially affecting USP10. Further investigation is needed to elucidate the precise molecular mechanisms involved.

p53 is widely recognized as a critical tumor suppressor gene, with *circTP53* promoting the stabilization of p53 protein through its interaction with USP10. This suggests that *circTP53* may play a tumor‐suppressive role. However, qRT‐PCR assay and ISH staining indicate a close correlation between *circTP53* and poor prognosis in HNSCC. Specifically, the depletion of *circTP53* significantly reduces the viability of HNSCC cells (FaDu and TU686), suggesting that *circTP53* functions as an oncogene. To clarify this paradox, it is important to consider the pivotal promotional effects of p53 mutations in tumors, especially in HNSCC, where it is highly altered in over 70% of patients,^[^ ^37^ ^]^ as mutations inducing GOF, LOF, or DNE can all contribute to tumor progression, although the predominant effect driving this progression remains a matter of debate.^[^ ^29^ ^]^ Sequencing of the TP53 gene in FaDu and TU686 cells identified the R248L and P151S mutations, respectively, with mutation occurring at 248 and 151 being common mutation sites of GOF in various tumors, especially in HNSCC^[^ ^31^, ^32^, ^38^, ^39^, ^40^ ^]^ This finding supports our hypothesis that the high frequency of TP53 mutations in clinical samples contributes to the statistical association between *circTP53* and adverse prognosis in HNSCC. To further elucidate the relationship between *circTP53* and p53 status, we knocked down *circTP53* in cells with wtp53 (A549, HeLa, HCT116 p53^+/+^), mtp53 (MDA‐MB‐231), or p53‐null (H1299, HCT116 p53^−/−^). The results showed that *circTP53* suppressed cell viability in p53 wild‐type status, promoted cell viability in the p53 mutational context, and had no effect in p53 null background. Specifically, depletion of *circTP53* in p53‐null cells rescued wtp53 or mtp53 resulted in suppressed or promoted cell viability, respectively. This differential response underscores the typically oncogenic role of *circTP53* in the context of p53 mutations.

Moreover, our findings suggest that *circTP53* may promote tumor progression in patients with p53 mutations, while exhibiting a tumor‐suppressive effect in those with wtp53. Recent studies using genetically engineered pig models have revealed that TP53 mutations drive a tumor spectrum, with *circTP53* playing a crucial role in tumorigenesis.^[^ ^41^ ^]^ This duality presents compelling evidence for considering *circTP53* as a potential therapeutic target, particularly within the framework of personalized medicine that is tailored to p53 status. For patients with GOF mutations, treatment strategies may need to focus on reducing *circTP53* levels. Conversely, for patients with wtp53, direct administration of *circTP53* could be a viable option. Research findings suggest that single‐stranded circular RNA exhibits higher transfection efficiency compared to linear or double‐stranded plasmids48. Given the inherent stability of *circTP53*, local administration through endoscopic techniques like injection and spraying may represent effective delivery methods in patients with HNSCC. This finding may offer some assistance in addressing the issue of clinical drug ineffectiveness in patients with wtp53. *CircTP53* could potentially provide an alternative treatment option for wtp53 patients beyond traditional chemotherapy and surgery.

Currently, the mechanism(s) underlying mtp53 accumulation in tumors remain poorly understood. However, mtp53 accumulation in tumors plays a pivotal role in facilitating mtp53 GOF in tumorigenesis.^[^ ^29^ ^]^ Interestingly, our statistical analyses revealed a significant correlation between *circTP53* and p53 in expression, indicating that additional regulatory mechanisms may be involved. This correlation highlights the need for further investigation into the interplay between *circTP53* and p53, as well as their combined impact on tumor biology and patient prognosis. Elucidating these underlying mechanisms may provide novel insights into the accumulation of mtp53 and inform the development of more effective therapeutic strategies for HNSCC.

Despite over three decades of research on p53‐targeted therapeutics, no clinically viable drugs have been developed thus far. Some RNA‐based drugs, such as COVID‐19 and SARS‐CoV‐2 mRNA vaccines,^[^ ^42^, ^43^ ^]^ have swiftly and efficiently engaged in clinical intervention, showcasing unparalleled clinical efficacy and socio‐economic benefits owing to their high specificity, controllability, and low immunogenicity.^[^ ^44^, ^45^ ^]^ *CircRNAs*, characterized by their unique closed‐loop structure, generally exhibit relatively stable properties compared to linear RNAs, making them resistant to exonuclease‐mediated degradation and they can only be degraded under extreme circumstances by specific endonucleases, such as RNase L, G3BP1, and RNase P/MRP.^[^ ^6^, ^46^, ^47^ ^]^ Emerging evidence suggests *circRNAs* serve as promising biomarkers and potential therapeutic targets in cancer. Recent advances in *circRNA* research highlight their potential as therapeutic targets in human cancer. *CircRNAs* like *circTP53* may play a key role in tumor progression, making them promising candidates for RNA‐based therapies. However, the off‐target effect remains one of the concerns. While *circRNAs* offer exciting therapeutic potential, careful consideration of off‐target effects is crucial for their safe and effective application. To minimize off‐target effects, we may use advanced whole‐genome sequencing technology and more robust in vivo and in vitro experiments to identify the specific targets for *circTP53* before the application of *circTP53‐*based therapy in clinical trials. To further enhance targeted precision, we might develop highly *circTP53*‐specific RNA therapies, optimize nanoparticle‐based *circTP53*‐specific delivery, use the modified oligonucleotides to reduce off‐target binding, and employ advanced screening for unintended interactions.^[^ ^48^, ^49^, ^50^, ^51^ ^]^ Particularly in early tumor detection, their longer half‐life and ability to be released into bodily fluids, such as exosomes, facilitate convenient detection. For HNSCC patients, combining techniques like throat swabs and endoscopy enables the acquisition of suitable samples for testing. Additionally, employing digital PCR technology offers sensitive and reliable detection, aiding in treatment decisions.

In this study, we have provided valuable insights into the role of *CircTP53*/USP10/p53 signaling axis in HNSCC, while this study also has some limitations. Although our findings found a novel function of *circTP53* in HNSCC progression, this study predominantly relied on in vitro cell line experiments and in vivo xenograft models. Although these models are widely used in experiments on cancer research, they may not fully replicate the complexity of human tumors, particularly in terms of the tumor microenvironment and immune responses. Further validation in more complex systems is needed to confirm the functional relevance of our findings in HNSCC. Furthermore, our sample size of study HNSCC patients is relatively small, and a larger patient cohort is needed to validate our findings on the function of *circTP53* in HNSCC. Finally, given the established role of HPV in HNSCC development, future studies should take tumor HPV status into consideration to better understand the role of *circTP53* in HNSCC carcinogenesis and progression.

## 4 Experimental Section

### Cell Lines, Antibodies, and Reagents

All human cell lines used in this study were from the American Type Culture Collection (ATCC). These cell lines were authenticated by STR locus analysis and tested for mycoplasma contamination. H1299 cells were maintained in RPMI 1640 (22400089, Gibco), HCT116 cells were maintained in McCoy's 5A (16600082, Gibco) and all other cells were maintained in DMEM (10 569 044, Gibco), supplemented with 10% FBS (HyClone) in a 37 °C incubator with 5% (v/v) CO2. The TP53 mutation status of all cell lines used in this study was as follows: FaDu (R248L), Tu686 (P151S), MDA‐MB‐231 (A280L), HeLa (wild‐type), H1299 (p53 null), HCT116−/− (p53 null), A549 (wild‐type), and HCT116+/+ (wild‐type). Primary antibodies used for western blot, immunoprecipitation, RIP, and immunofluorescence are listed in Table S1 (Supporting Information).

### Human Tissue Samples

HNSCC and adjacent nontumorous tissue samples were freshly resected from 150 patients at the Qilu Hospital of Shandong University from September 2016 to December 2018 and 42 patients from December 2023 to December 2024. Among them, 132 samples were used for RNA extraction and qPCR experiments, and 60 samples were fixed and embedded in paraffin for immunohistochemistry (IHC) and immunofluorescence (IF) analyses. The present study was approved by the Ethics Committee of Shandong University, and written informed consent was obtained from all patients (Ethical approval number: KYLL‐2020(KS)‐320).

### Plasmids and Cloning Strategies

The *circTP53*/USP10 shRNA or ctr (sh*circTP53*‐1/‐2; shUSP10‐1/‐2) was amplified and subcloned into the plasmids Lentiviral Interference Vector LV‐3 (pGLVH1/GFP+Puro, C06003, genepharma). The pLC5‐ciR, and pLC5‐ciR‐*circTP53* were purchased from GRNESEED (GS0108). *CircTP53*, USP10, or p53 and mutations of these molecules were inserted into these plasmids. The pmirGLO plasmid was purchased from promega (E1330).

### qRT‑PCR

The total RNA was synthesized into cDNA with PrimeScript RT Reagent Kit (RR037A, Takara) in accordance with the manufacturer's protocols. The cDNA was amplified with TB Green Premix Ex Taq (RR820A, Takara) on the LightCycler 480 Instrument II (Roche). The expression of *circRNA* and mRNA was determined by ­2^–ΔΔCT^ and normalized by β‐actin. The threshold for low/high expression levels was determined based on the median of *circTP53* expression value. The primers used in the study are listed in Table S1 (Supporting Information).

### RNA In Situ Hybridization (ISH)

ISH was conducted with a digoxin‐labeled probe specific for *circTP53* to evaluate the expression of *circTP53* on tissue microarrays which contained 60 HNSCC tissues and 60 normal tissues collected from Qilu Hospital. Briefly, the tissue microarrays were dewaxed and rehydrated, then digested with proteinase K and followed by hybridization with the above‐mentioned *circTP53* probe at 45 °C overnight. After that, the tissues were incubated with biotin‐conjugated antibodies against digoxin at 4 °C overnight, and then stained with DAB. The expression of *circTP53* was quantified by multiplying the scores of the intensity of positive staining (strong = 3, moderate = 2, weak = 1, and negative = 0) and the percentage of positive‐stained cells (>75% = 4, 51–75% = 3, 26–50% = 2, ≤25% = 1). The samples were defined as low or high‐expression groups by the mean of ISH scores.

### Northern Blot

Northern blotting was performed with the DIG Northern Starter Kit (12039672910, Roche) according to the manufacturer's instructions with minor modifications. Briefly, the DNA template used for the in vitro synthesis of probes labeled with digoxigenin to detect *circTP53* or linear *TP53* mRNA was generated by PCR, and the primers are listed in Table S1 (Supporting Information). Ten milligrams of total RNA with or without RNase R digestion was resolved on 2% agarose gels prepared with formaldehyde before transfer to a Hybond‐N+ membrane (Solarbio) by capillary transfer, and RNA was then fixed to the membrane through UV crosslinking (200 000 mJ cm^−2^ at 265 nm). Hybridization was performed at 68 °C for 6 h with a biotin‐labeled oligonucleotide probe. The membranes were blocked in blocking buffer for 30 min, and then incubated with antibody solution for 30 min with gentle shaking. The membranes were washed three times with washing buffer, incubated with detection solution for 5 min, and exposed to X‐ray film. GAPDH was used as an internal control.

### Cytosolic/Nuclear Fractionation

The NE‐PER Nuclear and Cytoplasmic Extraction Reagent Kit (#78833, Thermo) was used. Briefly, cells were harvested with trypsin‐EDTA, centrifuged at 500 × *g* for 5 min, washed with PBS, and resuspended in ice‐cold Cytoplasmic Extraction Reagent I (CER I). After vortexing, ice‐cold Cytoplasmic Extraction Reagent II (CER II) was added, followed by a 5‐second vortex. The mixture was centrifuged at ≈16 000 × *g* for 5 min to obtain the cytoplasmic extract. The nuclear pellet was resuspended in ice‐cold Nuclear Extraction Reagent (NER), vortexed for 40 min, and centrifuged at ≈16 000 × *g* for 10 min to obtain the nuclear extract.

### Cell Proliferation, Cell Apoptosis Assays

The growth curves of BC cells were obtained using Cell Counting Kit‐8 (CK04, Dojindo) according to the protocols of the manufacturer. As to colony formation assays, HNSCC cells (1000/well) were inoculated into 60 mm Cell Culture Dishes and cultured for two weeks, followed by fixing and staining with 0.5% crystal violet. Cell apoptosis assays were analyzed on a flow cytometer (Becon Dickinson FACS Calibur, NY, USA) with PI staining and Dead Cell Apoptosis Kit (V13242, Thermo), respectively.

### Transwell Invasion Assay

Cells were treated as required and then reseeded into the upper chambers (3415r, Costa), with a medium containing a higher concentration of FBS placed in the lower chambers. After 48 h, the cells remaining on the membrane of the upper chambers were fixed with 4% paraformaldehyde (P1110, Solarbio) and stained with crystal violet staining solution (C0121, Beyotime). For the invasion assay, the upper chambers were coated with Matrigel (C0371, Beyotime) at 37 °C before seeding the cells.

### Fluorescence In Situ Hybridization (FISH) and Immunofluorescence (IF) Co‑Staining

The coverslips seeded with cells were hybridized with Cy3‐labeled probes (5′‐GGC CTT TGG CTC CTC TGT CCA AAC CAG TAT TAA GTA AGGT‐Cy3‐3′) (Geneseed) targeting the junction site of *circTP53* using fluorescent in situ hybridization kit (C10910, RiboBio) according to the manufacturer's protocols. Then, the coverslips were incubated with antibodies specific for USP10 (1:200, HPA006731, Sigma–Aldrich) at 4 °C overnight and FITC‐conjugated secondary antibodies at 37 °C for 1 h, followed by counterstaining with DAPI.

### RNA Antisense Purification (RAP)

The biotin‐labeled probes targeting the junction site of *circTP53* (5′‐ACA TCT TGT TGA GGG CAG GGG AGT ACT GGA GTG AGC CCTG‐Biotin) were synthesized by Sangon Biotech. RAP was performed using the RNA Antisense Purification (RAP) Kit (Bes5103‐3, BersinBio) following the manufacturer's instructions. Afterward, the potential interacting proteins were evaluated with a western blot or mass spectrometry analysis (C500021, BersinBio).

### Dual‑Luciferase Reporter Assay

Partial sequence of Intron9 of *circTP53* sequence subcloned into pmirGLO (E1330, Promega) to construct luciferase reporter vectors. Cells were co‐transfected with USP10‐WT or USP10‐MT plasmids and a partial sequence of intron9 reporter constructs with renilla luciferase. The luciferase activity of the reporters was detected with Dual‐Luciferase Reporter Assay System (E1910, Promega).

### Xenograft Mouse Model

The four‐week‐old female BALB/c nude mice were (Beijing Vital River) housed under the standard conditions at the NHC Key Laboratory of Otorhinolaryngology. FaDu cells (1 × 10^7^) were subcutaneously inoculated into the dorsal flanks of the randomly grouped nude mice. The tumor size of each mouse was monitored and calculated by length × width^2^ × 0.5. Four weeks later, the tumor‐bearing mice were sacrificed, then excised and weighed. The animal testing procedures have been approved by the Code of Ethics and reviewed and implemented according to the guidelines of the Animal Care and Use Committee of The First Clinical Medical School, Shandong University (number: KYLL‐2023(ZM)‐150).

### Immunohistochemistry

Paraffin‐embedded sections were dewaxed and rehydrated, then incubated with primary antibodies specific for USP10 (1:200, HPA006731, Sigma–Aldrich) at 4 °C overnight and biotin‐labeled secondary antibodies at 37 °C for 1 h. The slides were then stained with DAB and hematoxylin, followed by photographing under a microscope (Leica, Wetzlar, Germany).

### Western Blot

Precast gels were purchased from GenScript (M42015C, M41215C). Lysates were loaded onto the gels and run with Tris‐MOPS‐SDS running buffer (M00138, GenScript) in electrophoresis chambers. Total proteins of FaDu and Tu686 cells were extracted with RIPA lysis buffer containing PMSF and complete Protease Inhibitor EASYpacks (04693132001, Roche) subjected to SDS‐PAGE, then transferred onto NC membranes (Millipore, Billerica, MA, USA). The membranes were blocked with 5% skimmed milk and incubated with primary antibodies specific for USP10 (1:1000, #8501 Cell Signaling), p53 (1:1000, sc‐126, Santa Cruz), Bax (1:1000, 50599‐2‐Ig, Proteintech), p21 (1:1000, 10355‐1‐AP, Proteintech) or β‐actin (1:1000, sc‐47778, Santa Cruz) at 4 °C overnight and HRP‐conjugated secondary antibodies at room temperature for 2 h. The bands were finally visualized using an infrared laser imaging system called Odyssey (LI‐COR Biosciences).

### Co‑Immunoprecipitation (Co‑IP)

Co‐immunoprecipitation was executed with antibodies specific for USP10 (1:200, HPA006731, Sigma–Aldrich) or p53 (1:200, sc‐126, Santa Cruz), normal mouse IgG2a Control (sc‐3878, Santa Cruz) or Normal Rabbit IgG (#2729 Cell Signaling), and Pierce Classic Magnetic IP/Co‐IP Kit (88804, Thermo). In Brief, cells were harvested and lysed with IP lysis/wash buffer supplemented with protease inhibitor cocktail for 20 min on ice, then centrifugated at 14 000 × *g* for 20 min. The supernatant was collected and incubated with antibodies (5 µg) on a rotator at 4 °C overnight. After that, 25 µL Protein A/G Magnetic Beads were pre‐washed and incubated with the lysate/antibody mix for 4 h at 4 °C. The beads were collected with a magnetic stand and then washed with IP lysis/wash buffer and ultra‐pure water. The proteins were eluted with 100 µL of Lane Marker Sample Buffer and heated at 100 °C for 10 min, then followed by western blot.

### RNA Immunoprecipitation (RIP)

RNA immunoprecipitation assay was performed with antibodies specific for USP10 (1:200, HPA006731, Sigma–Aldrich), normal Rabbit IgG (#2729 Cell Signaling), and RNA Immunoprecipitation Kit (P0101, Geneseed) according to the recommended conditions. The co‐precipitated RNAs and USP10 proteins were detected with qRT‐PCR and western blot, respectively.

### Statistical Analysis

Prism GraphPad software v9.0 was used for analysis. Each experiment was performed in triplicate, and the data are shown as the mean ± SD, unless otherwise stated. Associations of *circTP53* expression with clinicopathological characteristics were analyzed by chi‐square Fisher's exact tests. Associations of *circTP53* expression with the prognosis of HNSCC patients were analyzed by Kaplan–Meier analysis with the log‐rank test. *p*‐Values of 0.05 or less were considered significant.

### Ethics Approval

The present study was approved by the Ethics Committee of Shandong University, and written informed consent was obtained from all patients (Ethical approval number: KYLL‐2020(KS)‐320). The animal study was performed following the guidelines of the Shandong University Animal Care and Use Committee (number: KYLL‐2023(ZM)‐150).

## Acknowlegements

This work was supported by the National Natural Science Foundation of China (No. 82071918 and No. 82471149) and the Natural Science Foundation of Shandong Province (ZR2021QC062). The authors would like to thank Prof. Bert Vogelstein (Johns Hopkins Medical School, Baltimore, USA) for his kind donation of the HCT116 p53^+/+^ and HCT116 p53^−/−^ cells. The authors also thank Dr. Xiao Zhao and Dr. Andrew G. Sikora for their reviewing and kind help with editing the manuscript.

## Conflict of Interest

The authors declare no conflict of interest.

## Author Contributions

Y.W. and F.C. contributed equally to this work. D.L. and F.C. conceived and designed the experiments; Y.W. performed the experiments and wrote the manuscript; Z.L., C.D., X.S., S.W., and J.Z. made substantial contributions to the analysis of clinical biomarker data and provided support with experimental techniques; S.C., D.W., W.L., and Y.Q. provided support with clinical samples; and F.C. revised the manuscript. All authors contributed to the article and approved the submitted version.

## Supporting information



Supporting Information

## Data Availability

Data sharing is not applicable to this article as no new data were created or analyzed in this study.
