# Supplementary material for: CircTP53/USP10/p53 signaling Axis as a Novel Regulator of Progression and Prognosis of Head and Neck Squamous Cell Carcinoma
Source: Adv Sci (Weinh). 2025 Jun 4;12(30):e14961. doi: 10.1002/advs.202414961 (PMC12376528; doi:10.1002/advs.202414961)

**
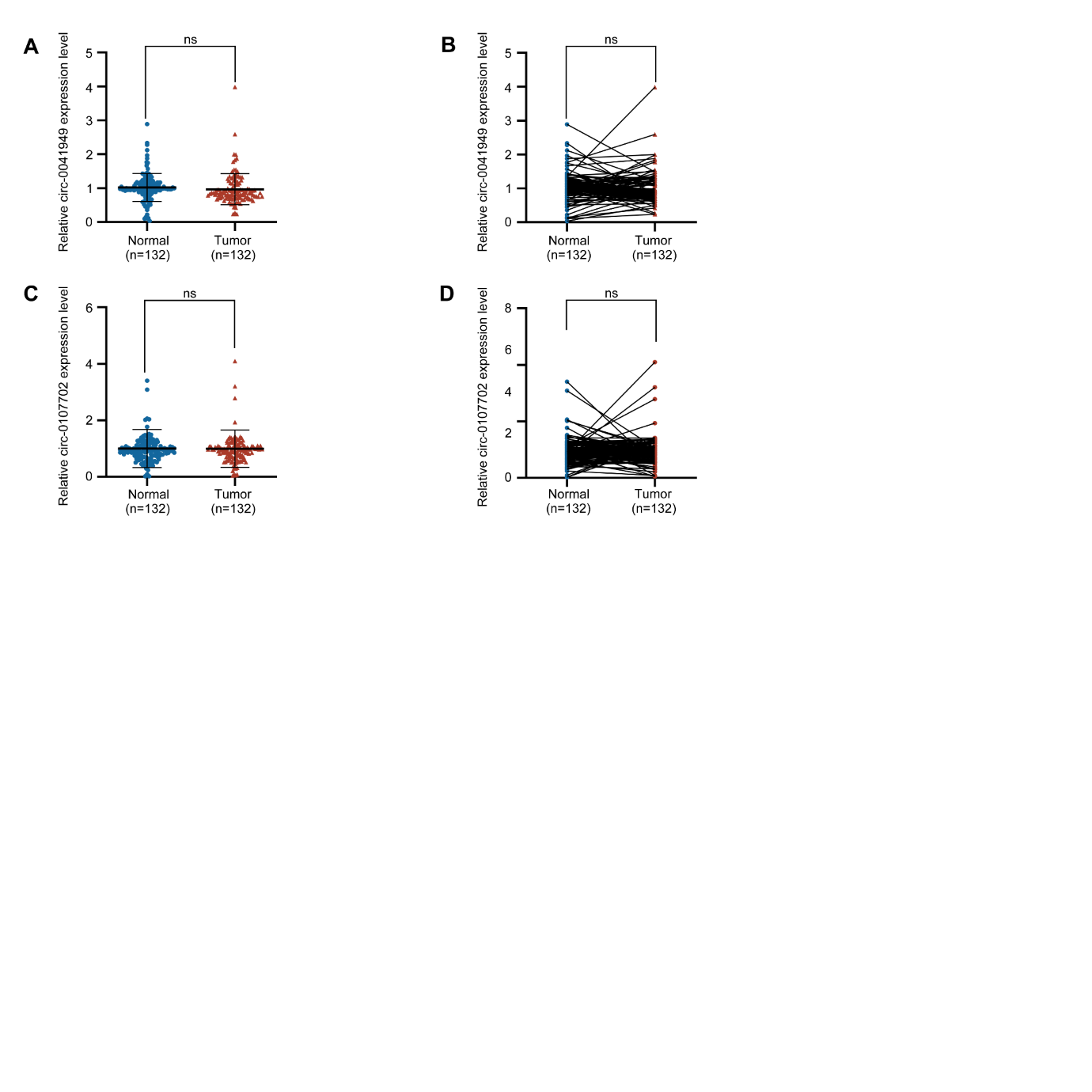
**

**Supplementary Fig. 1.** The relative levels of *circRNAs* from the TP53 gene expression

**A-D**. The qRT-PCR assay showing the relative levels of *hsa-circ-0041948* and *hsa-circ-0107702* (normalized to β-actin) in the peritumor and tumor tissues of HNSCC (n = 132). **, P < 0.01. ns: no statistical significance.


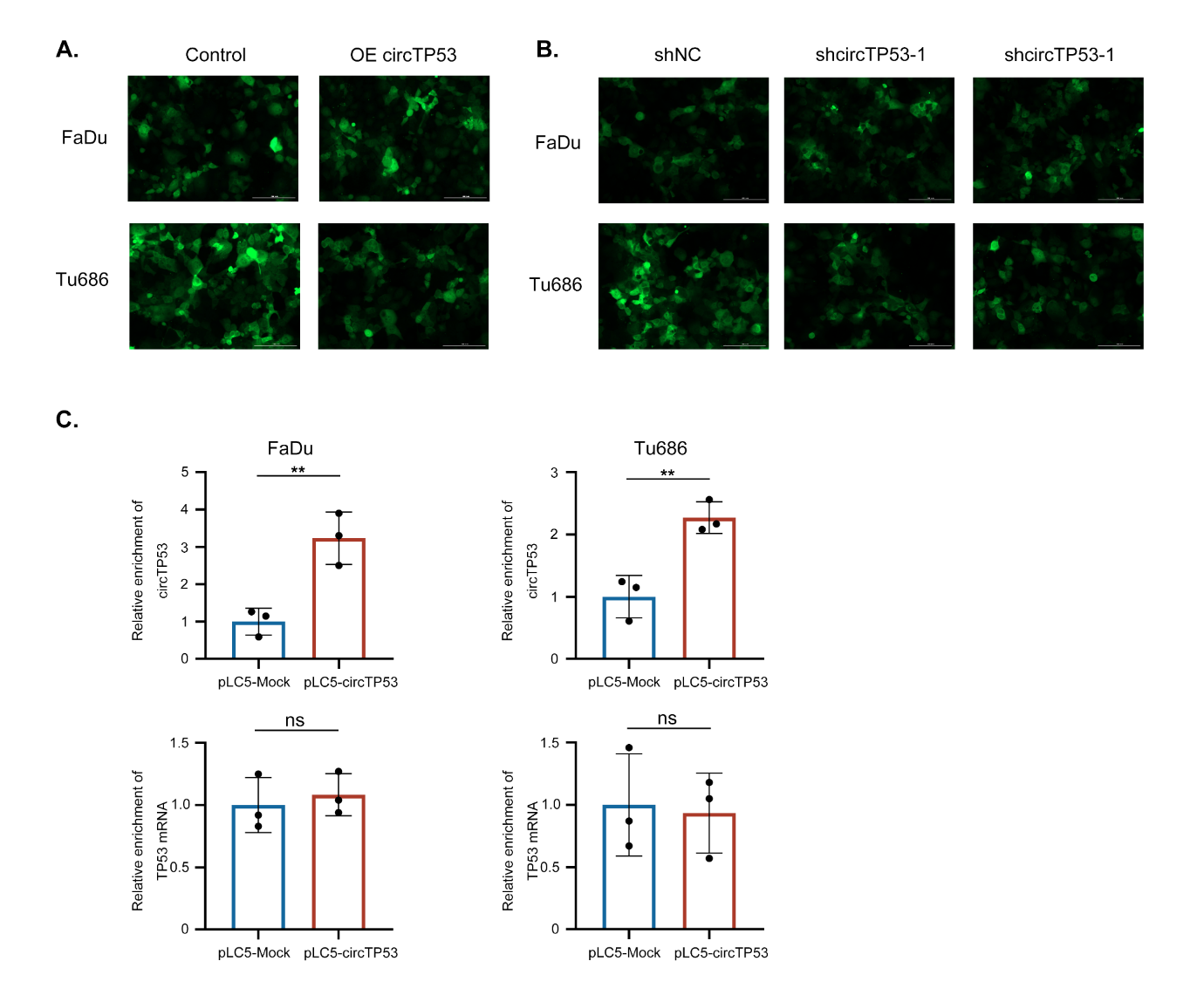


**Supplementary Fig. 2.** Validation of knockdown and overexpression cell lines.

pGLVH1/GFP+Puro, *circTP53* shRNA (sh*circTP53*-1/ sh*circTP53*-2), pLC5-Mock, and pLC5-circTP53 (OE circTP53) **A**. *circTP53* plasmids were stably transfected into both HNSCC cells to establish stable overexpression cell lines. Quantification of GFP tags fluorescence verification was shown. **B**. shNC and shcircTP53-1/ shcircTP53-2 were transfected into both HNSCC cells to establish negative control and stable circTP53 knockdown cell lines. Quantification of GFP tags fluorescence verification was shown. **C**. The qRT-PCR was used to show increased expression of *circTP53* (*p* < 0.05) and unchanged *TP53* mRNA levels in FaDu and Tu686 cells with transfected with OE circTP53. (Data are presented as the mean ± SEM, ns > 0.05, *p < 0.05, **p < 0.01). (All dots on the bar chart represent the mean values of each repeated experiment).


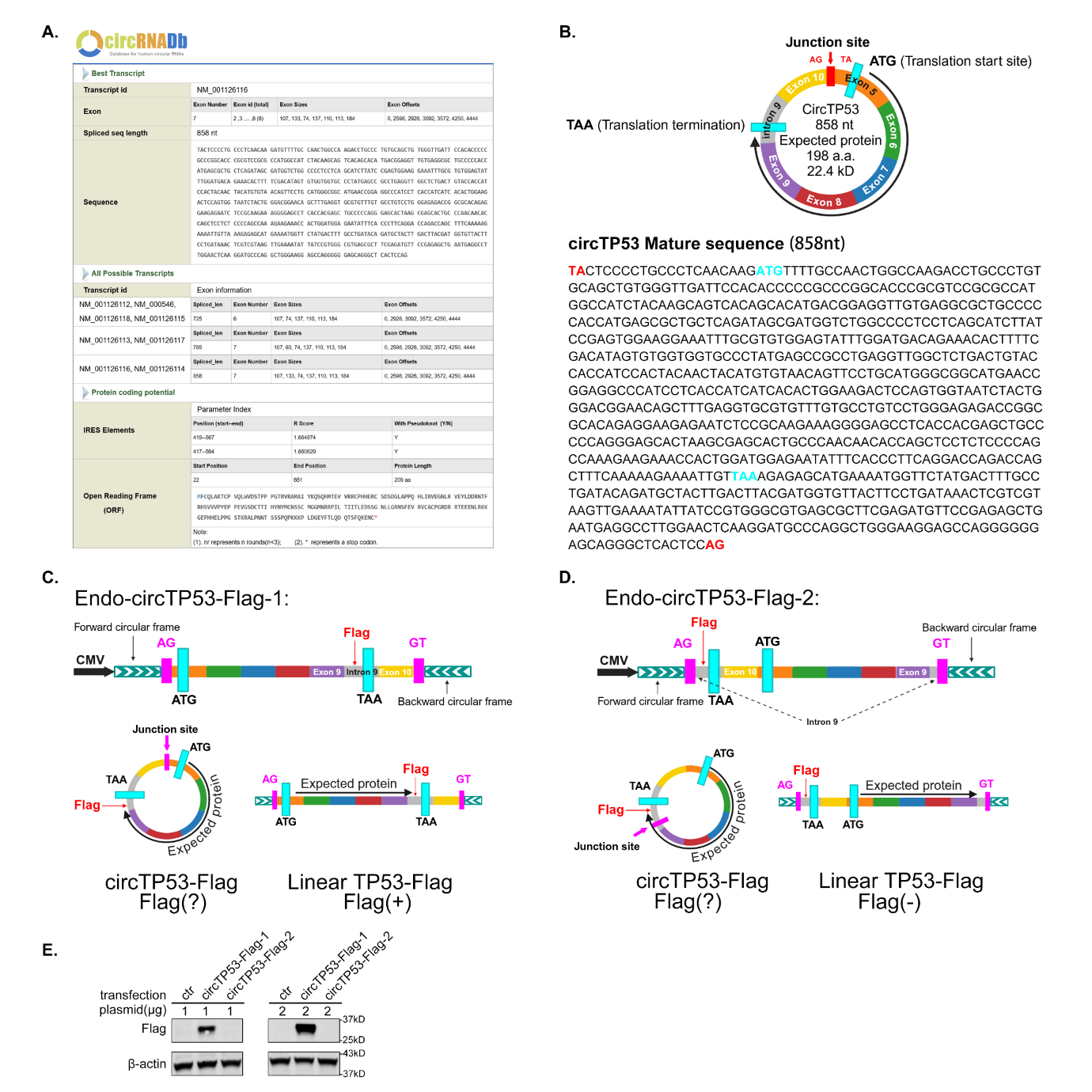


**Supplementary Fig. 3.** *circTP53* may not be translated into protein

**A**. Prediction of the potential encoded protein of *circTP53* on the *circRNA*Db website. **B**. Pattern diagram of *circTP53* expression protein, marked with open reading frame, start codon and stop codon. **C and D**. Schematic diagram of circular and linear transcripts of the Plc5-ciR-*circTP53*-Flag-1 and Plc5-ciR-*circTP53*-Flag-2 vector. **E**. Plasmid-encoded protein: the overexpression plasmid with the modified junction site cannot encode protein. (Data are presented as the mean ± SEM, ns > 0.05, *p < 0.05, **p < 0.01).


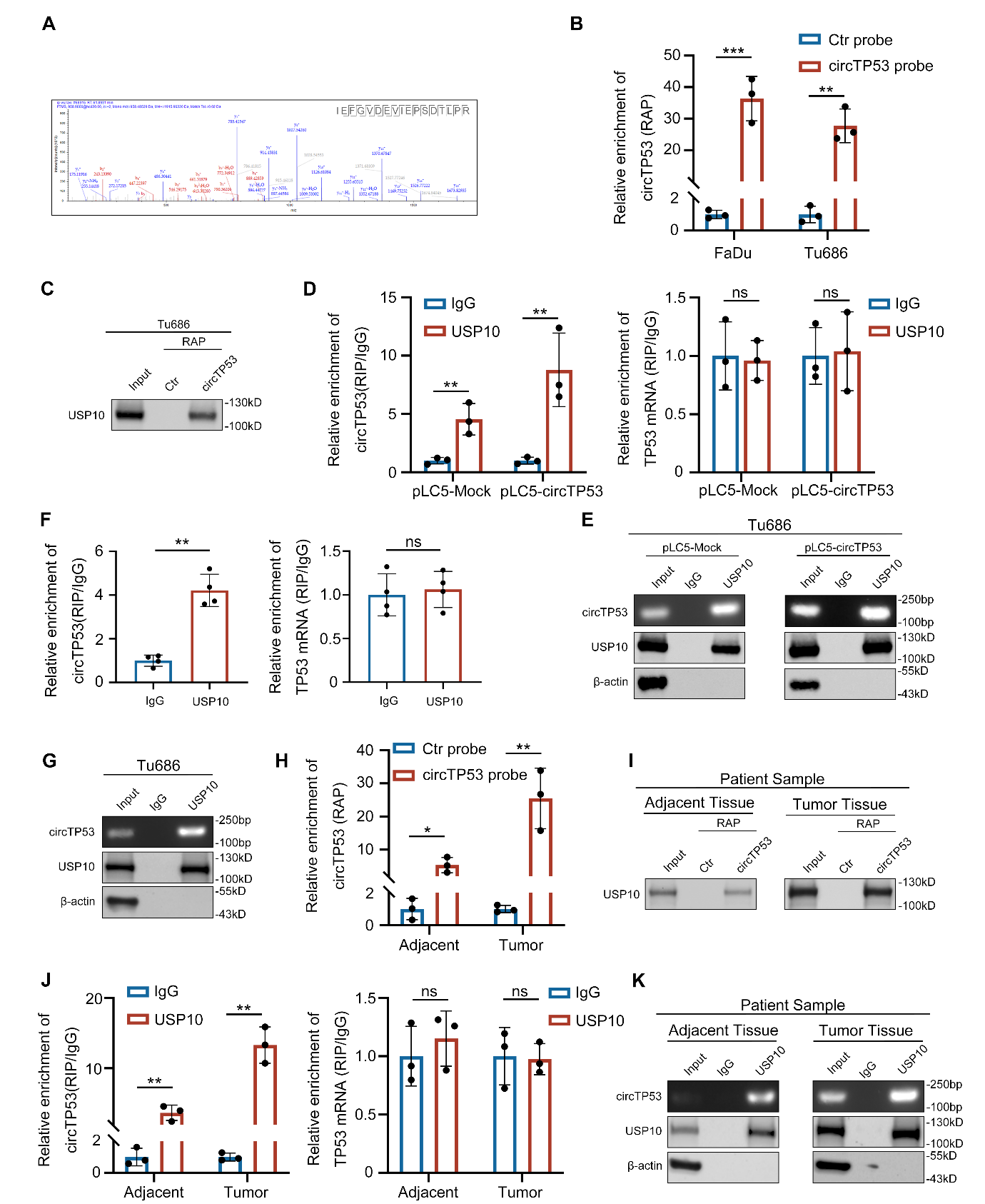


**Supplementary Fig. 4.** *CircTP53* interacts with usp10 and stabilized by usp10 in HNSCC cell lines and patient samples.

**A.** Unique peptides of USP10 identified by mass spectrometry analysis. **B**. The qRT-PCR detection of *circTP53* pull-down efficiency in the RAP assay in HNSCC cell lines. **C**. The interaction between *circTP53* and USP10 was verified by RAP assay and WB in Tu686 cells. **D-G**. Endogenous and exogenous RIP assays were carried out in Tu686 cells using anti-USP10 and IgG control, followed by qRT-PCR of *circTP53* and *TP53* mRNA. **H.** The qRT-PCR detection of *circTP53* pull-down efficiency in the RAP assay in HNSCC Patient samples. **I.** The interaction between *circTP53* and USP10 was verified by RAP assay and WB in adjacent tissue and tumor tissue. **J-K**. The RIP assays were carried out in adjacent and tumor tissues using anti-USP10 and IgG control, followed by qRT-PCR of *circTP53* and *TP53* mRNA. (the data presents the mean ± SEM, ns > 0.05, *p < 0.05, **p < 0.01) (All dots on the bar chart represent the mean values of each repeated experiment).


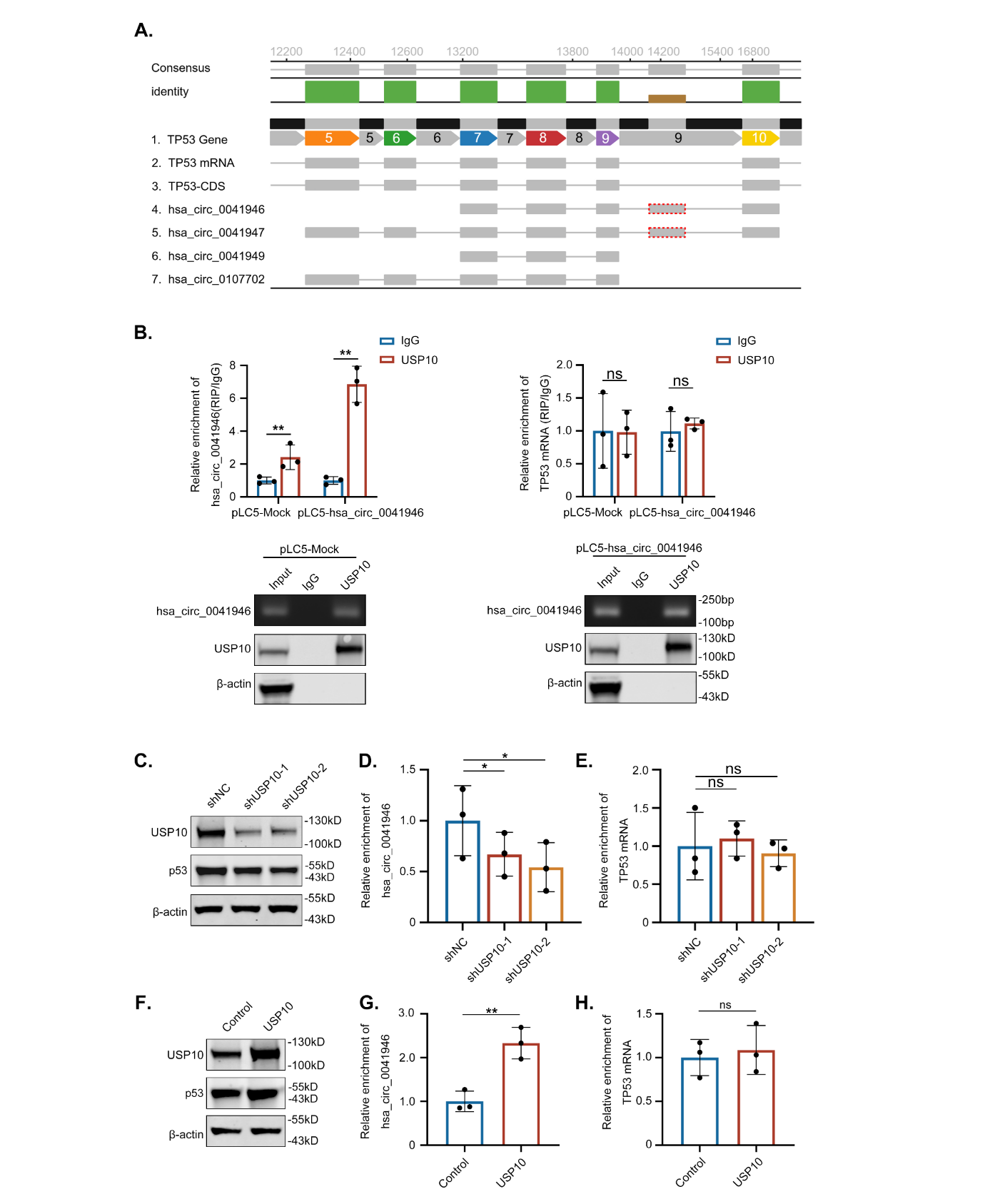


**Supplementary Fig. 5.** *hsa-circ-0041946* interacts with usp10 and stabilized by usp10

**A**. Schematic diagram comparing *circRNAs* originating from the *TP53* gene sequences to p53 mRNA obtained from NCBI. **B**. RIP assays of exogenous were carried out in FaDu cells under indicated conditions using anti-USP10 and IgG control, followed by qRT-PCR of *hsa-circ-0041946* and *TP53* mRNA. **C-E**. Western blot analyses of FaDu cells after knockdown of USP10 with indicated antibodies, followed by qRT-PCR of *hsa-circ-0041946* (D) and *TP53* mRNA (E). **F-H** Western blot analyses of FaDu cells after overexpression of USP10 with indicated antibodies, followed by qRT-PCR of *hsa-circ-0041946* (G) and *TP53* mRNA (H). (Data are presented as the mean ± SEM, ns > 0.05, *p < 0.05, **p < 0.01) (All dots on the bar chart represent the mean values of each repeated experiment).


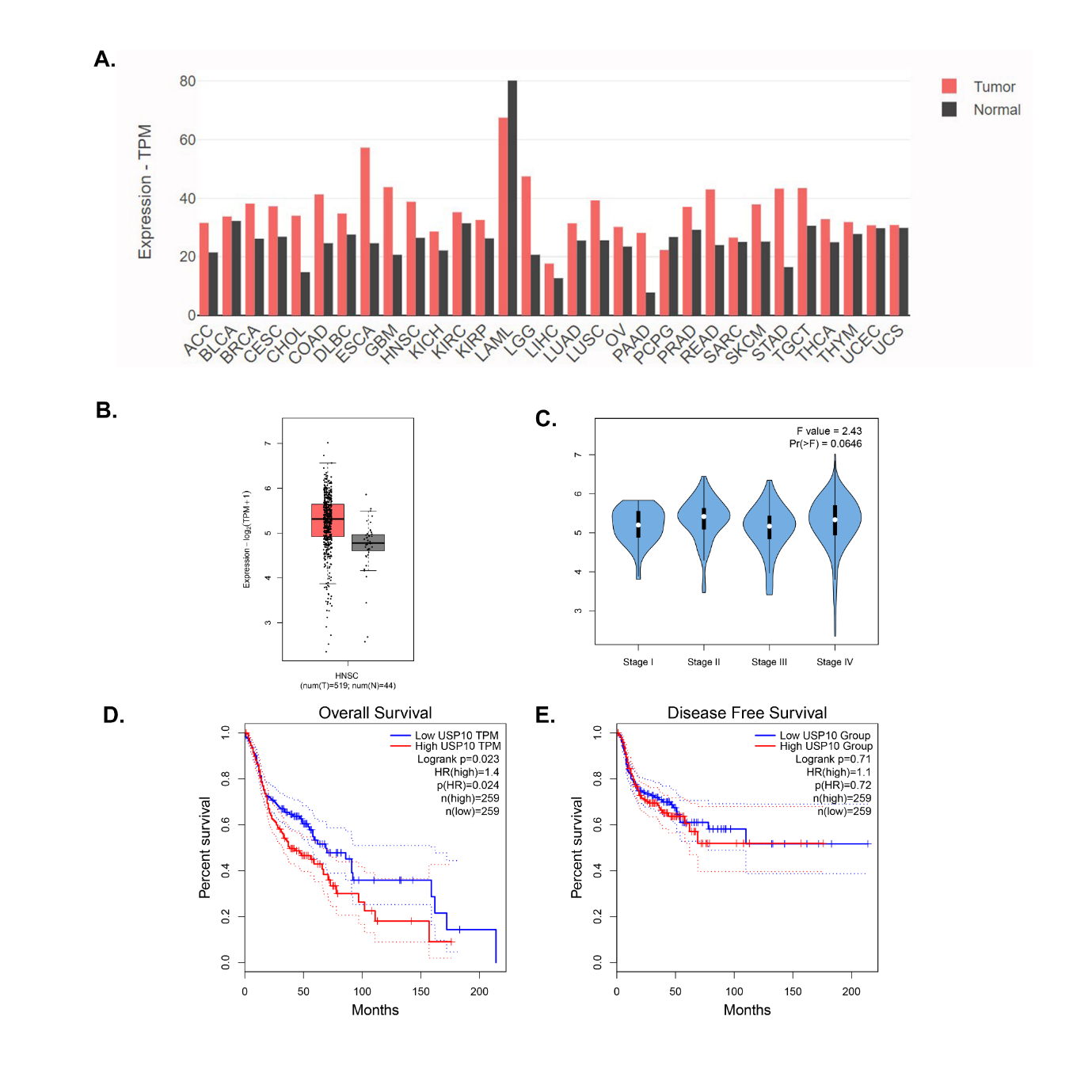


**Supplementary Fig. 6.** The expression of USP10 was higher in HNSCC tissues than in the adjacent tissues.

**A.** The expression pattern of USP10 was revealed in pan-cancer by analyzing the publicly GEPIA(2) database (http:// <http://gepia.cancer-pku.cn/>). **B.** The expression pattern of USP10 was revealed in HNSCC in GEPIA(2) database. **C**. The USP10 mRNA expression level for the patient characteristics of pathologic stage. **D**. Kaplan-Meier analysis of USP10 mRNA expression levels and OS (overall survival). E. Kaplan-Meier analysis of USP10 mRNA expression levels and DFS (Disease Free Survival)


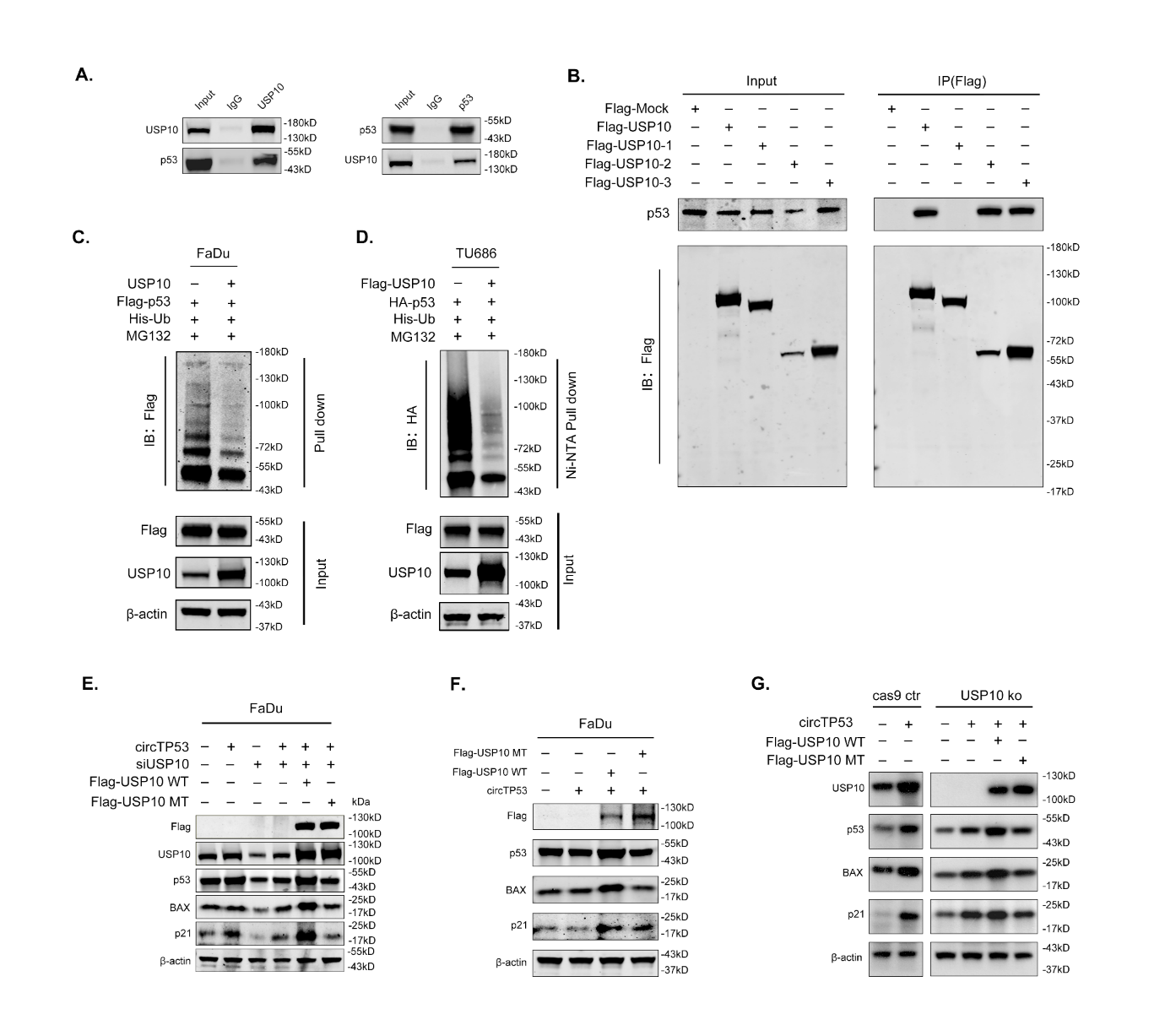


**Supplementary Fig. 7.** *circTP53* stabilizes p53 by stabilizing usp10 and promoting deubiquitination of p53 by usp10

**A**. Co-IP assays to analyze the direct interaction between USP10 and p53 in FaDu cells using anti-USP10 or anti-p53, respectively. **B**. Co-IP was applied in FaDu cells transfected with indicated full-length or truncated USP10 plasmids using anti-Flag. C and D. Ubiquitination of p53 was analyzed in MG132-treated HNSCC cells bearing Flag-Mock and Flag-USP10. **E**. Western blot analyses of FaDu cells after co-transfecting with USP10 WT or USP10 MT and *circTP53* showing the rescue of knockdown of USP10 with indicated antibodies. **F**. Western blot analyses of FaDu cells after co-transfecting with USP10 WT or USP10 MT and *circTP53* showing the p53 protein level with indicated antibodies. **G**. Western blot analyses of FaDu cells after KO USP10 showing the rescue of KO phenotype by *circTP53* with indicated antibodies.


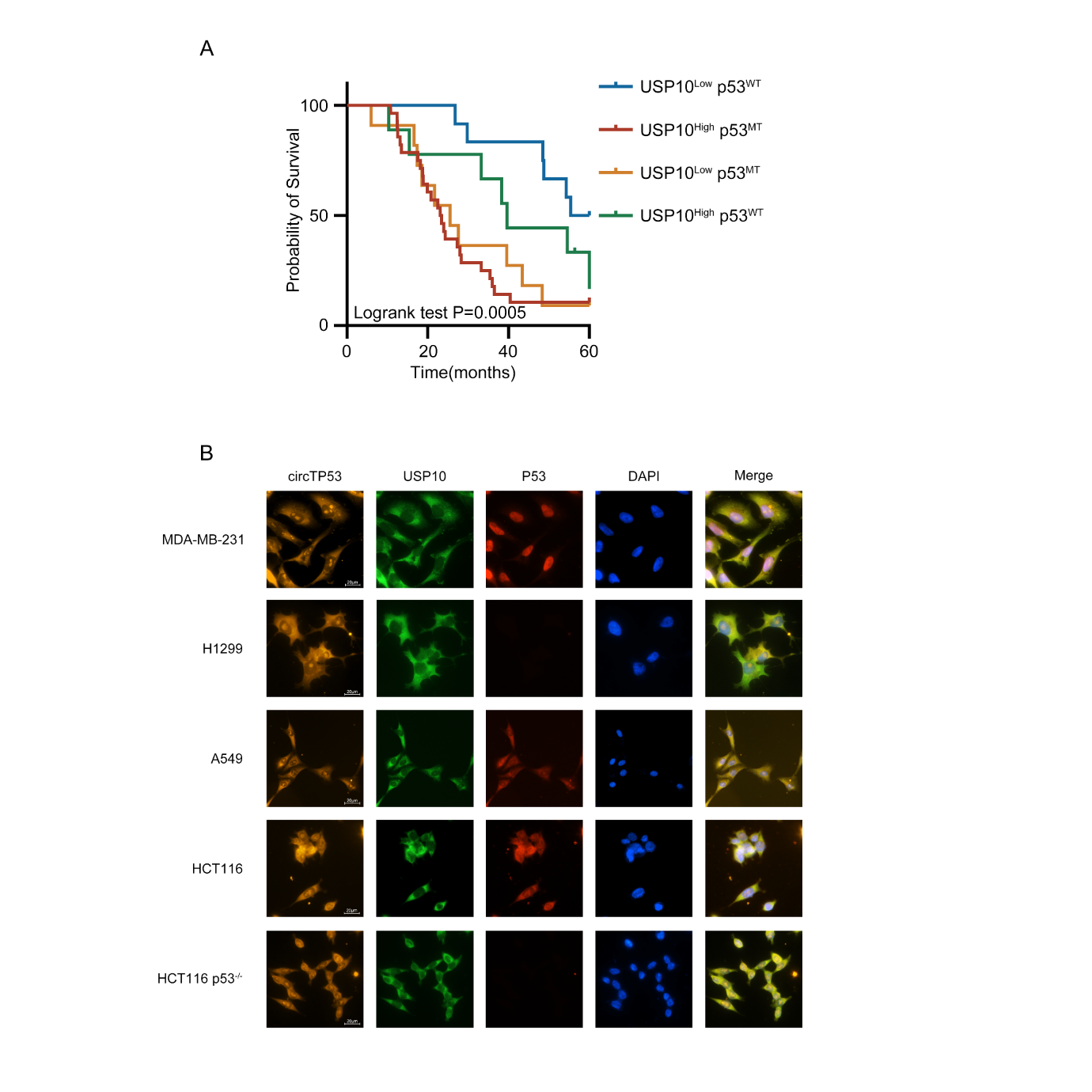


**Supplementary Fig. 8.** Survival analysis by expression of USP10 and MTp53 and cellular location of *circTP53*, USP10, and p53, respectively

**A**. High expression of USP10 and MTp53 are correlated with the lowest overall survival rate. Kaplan-Meier analysis was performed in four groups of HNSCC tumor tissues, with log-rank test, p = 0.0005. **B**. FISH and IF co-staining indicating the co-localization of *circTP53* (orange), USP10 (green) and p53 (red) in pan-cancer cells. Nuclear were stained with DAPI. Scale bar, 20 μm.


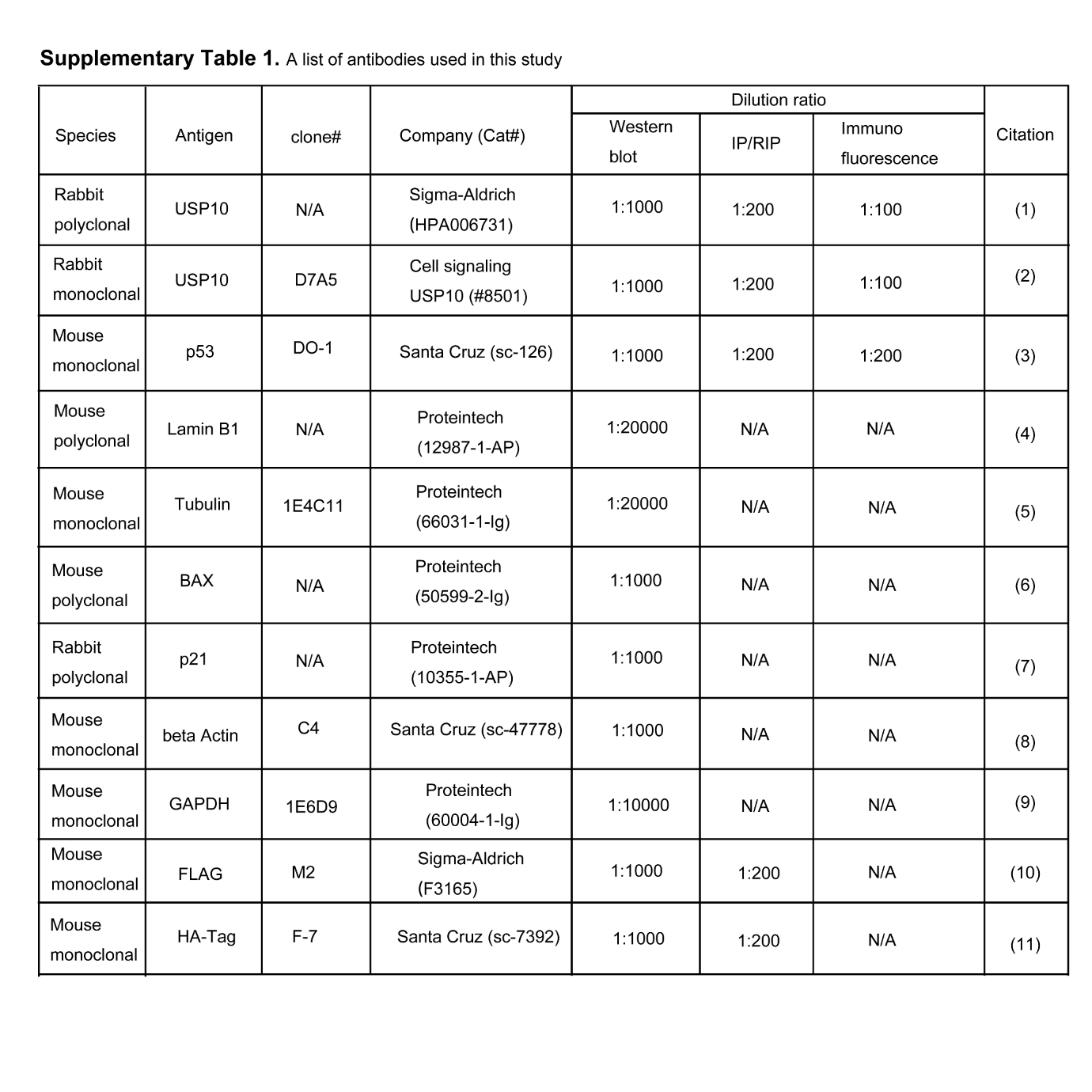


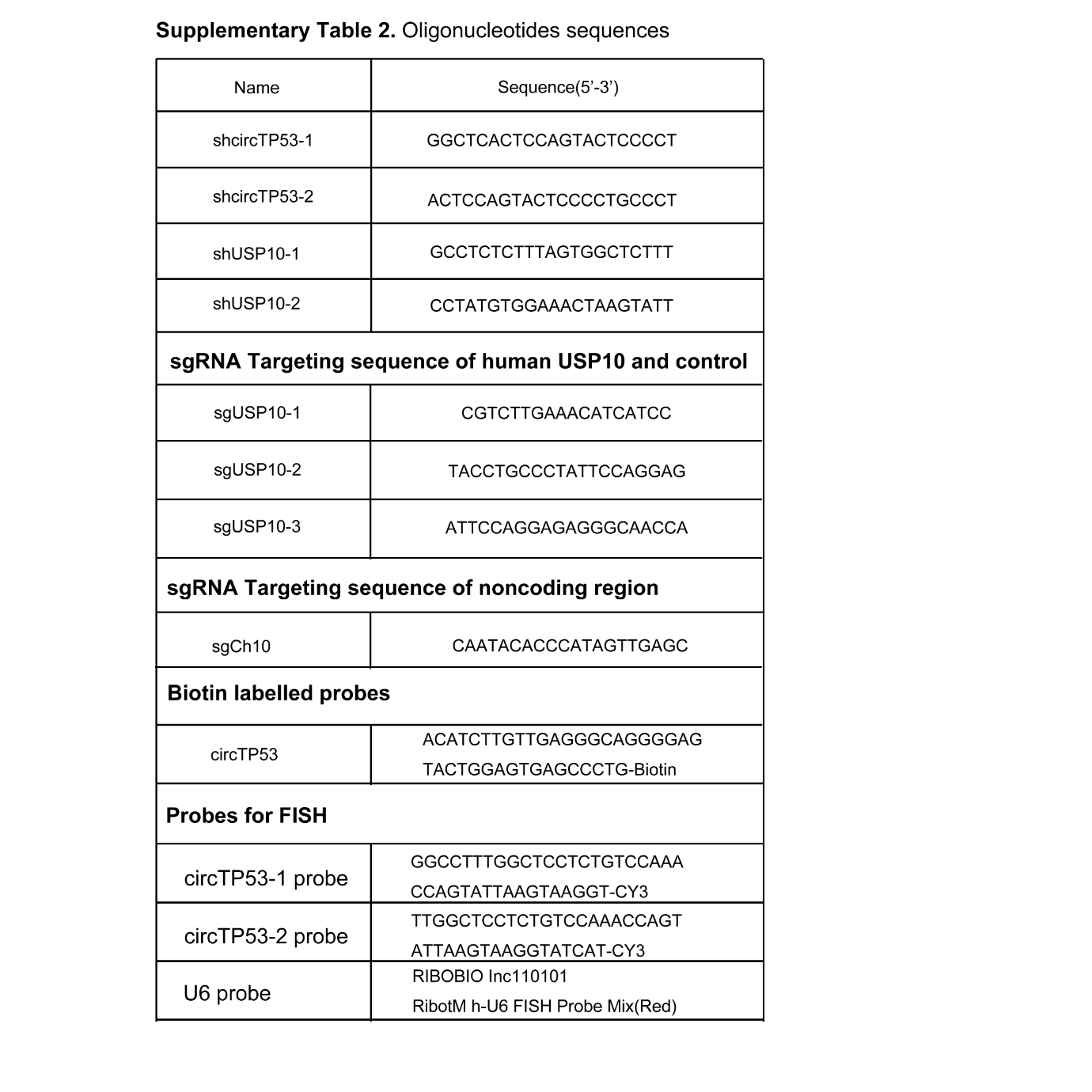


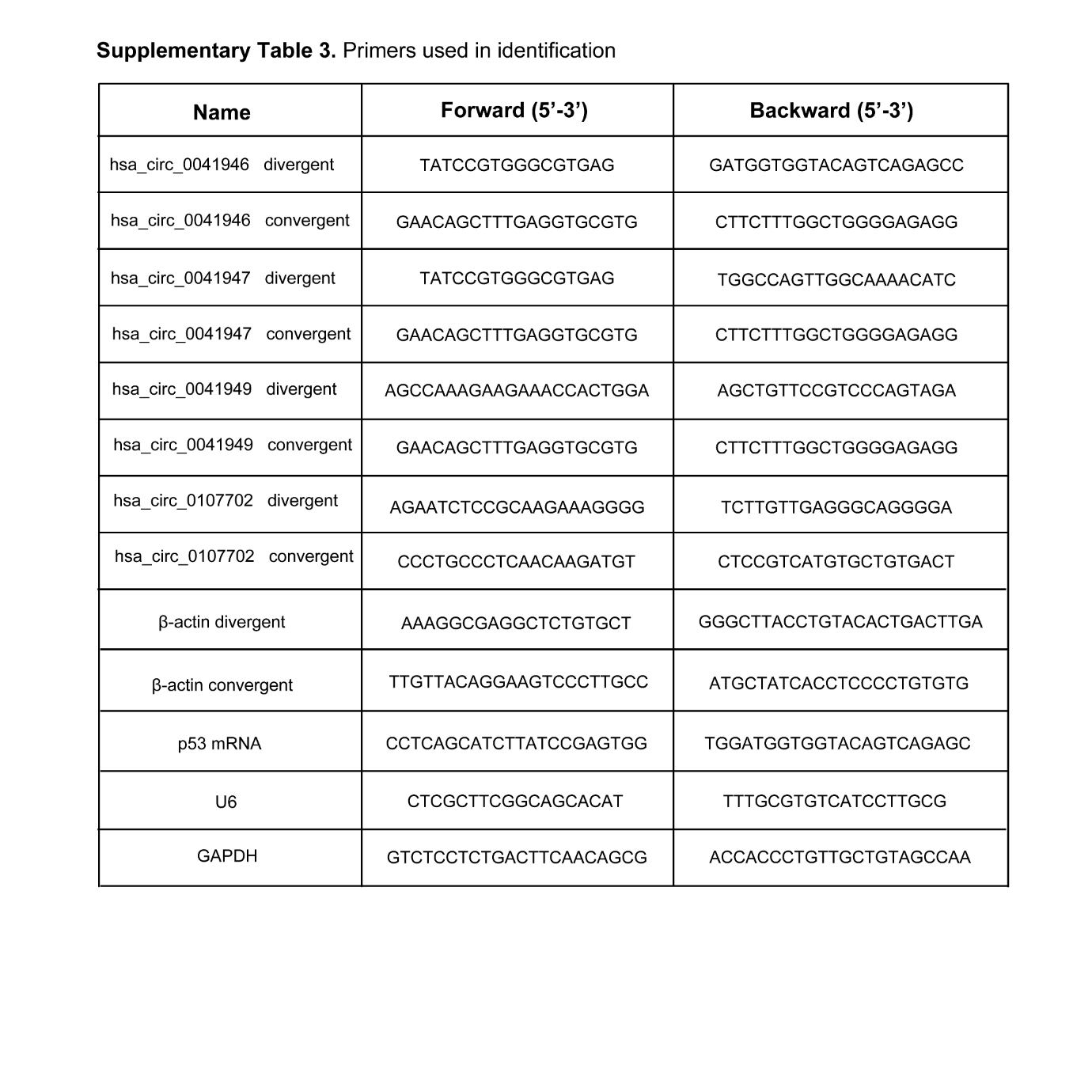

Supplement: Supplementary file 1 — Supporting Information [file ADVS-12-e14961-s001.docx]
